# Changes in perceptions of neighborhood environment and Cardiometabolic outcomes in two predominantly African American neighborhoods

**DOI:** 10.1186/s12889-019-8119-9

**Published:** 2020-01-14

**Authors:** Tiffany L. Gary-Webb, Natalie Suder Egnot, Alvin Nugroho, Tamara Dubowitz, Wendy M. Troxel

**Affiliations:** 1 Departments of Behavioral and Community Health Sciences and Epidemiology, University of Pittsburgh Graduate School of Public Health 130 DeSoto St, Rm 6135 Public Health Building, Pittsburgh, PA 15261 USA; 2 RAND Corporation, Pittsburgh, PA USA

**Keywords:** Neighborhood environment, Neighborhood change, Cardiometabolic health, African Americans

## Abstract

**Background:**

Perceived neighborhood characteristics, including satisfaction with one’s neighborhood as a place to live, are associated with lower obesity rates and more favorable cardiovascular risk factor profiles. Yet, few studies have evaluated whether changes in perceived neighborhood characteristics over time may be associated with cardiometabolic health indicators.

**Methods:**

Changes in perception of one’s neighborhood (2013–2016) were determined from a cohort of residents who lived in one of two low-income urban neighborhoods. Changes were categorized into the following: improvement vs. no change or worsening over the three-year time-period. Multivariable linear regression was used to measure the association between perceived improvement in each of the neighborhood characteristics with cardiometabolic outcomes (BMI, SBP, DBP, HbA1c, HDL-c) that were assessed in 2016, and compared with those who perceived no change or worsening of neighborhood characteristics. Models were adjusted for age, sex, income, education, marital status, physical function, neighborhood, and years spent in neighborhood. To examine potential sex differences, follow-up models were conducted and stratified by sex.

**Results:**

Among the 622 individuals who remained in the same neighborhood during the time period, 93% were African American, 80% were female, and the mean age was 58 years. In covariate-adjusted models, those who perceived improvement in their neighborhood safety over the time period had a significantly higher BMI (kg/m^2^) than those who perceived no improvement or worsening (β = 1.5, *p* = 0.0162); however, perceived improvement in safety was also significantly associated with lower SBP (mmHg) (β = − 3.8, *p* = 0.0361). When results were stratified by sex, the relationship between improved perceived neighborhood safety and BMI was only evident in females.

**Conclusions:**

These findings suggest that perceived neighborhood characteristics may impact cardiometabolic outcomes (BMI, SBP), but through differing pathways. This highlights the complexity of the associations between neighborhood characteristics and underscores the need for more longitudinal studies to confirm the associations with cardiometabolic health in African American populations.

## Introduction

Disproportionate rates of chronic disease among certain racial and ethnic groups in the United States (US) are well-documented. For instance, non-Hispanic Blacks and Mexican Americans have double the risk of diabetes compared to Non-Hispanic Whites, and 60% of US blacks have high blood pressure compared to 33% of US Whites [1–3]. The reasons for these disparities are complex and include individual and community-level factors. The neighborhood where one lives may be a potential factor contributing to these disparities, as African Americans and Hispanics are significantly more likely to live in disadvantaged neighborhoods with fewer resources compared to whites, even after adjusting for individual socioeconomic status (SES) [4].

Research aimed at understanding neighborhood-level determinants of health is rapidly expanding. Data has shown that residence in a disadvantaged neighborhood is associated with increased rates of obesity, diabetes, stroke, and cardiovascular disease (CVD) morbidity and mortality, as well as lower life expectancies [5–10]. These associations may be mediated through pathways involving physical activity, diet, and sleep [11–20]. However, the literature regarding the potential association between neighborhood characteristics and heart health remains limited for various reasons. First, much of the research has observed the association with health outcomes at a cross-sectional level, despite the dynamic nature of neighborhoods [14, 19, 21]. Prior studies also suffer from the use of convenience samples that threaten external validity and comparability to other studies.

Findings from the limited, existing longitudinal studies of neighborhood conditions and clinically relevant cardiometabolic outcomes in African-American populations are equivocal. The Atherosclerosis Risk in Communities (ARIC) Study, a multi-site cohort study with African American and white participants, examined neighborhood characteristics in relation to coronary heart disease CHD incidence and CVD mortality and found mixed results [22, 23]. While researchers found that neighborhood conditions were associated with increased incidence of CHD, they also found a lack of association between neighborhood conditions and CVD mortality among African Americans. Neighborhood characteristics in ARIC, however, were assessed via census data on socio-economic characteristics only and thus were limited in capturing the potentially dynamic nature of neighborhoods, as well as the geographic area that may be relevant. The Cardiovascular Health Study (CHS), another multi-site cohort study focused on following older adults, found no association between neighborhood SES and ischemic stroke among African Americans [24]. Lastly, the Jackson Heart study, which followed an African American cohort, measured multiple heart health outcomes including risk factors, subclinical measures and disease endpoints. Results indicated that neighborhood disadvantage, as measured by Census data, was associated with metabolic syndrome in African American women, and lack of perceived safety was associated with high glucose in both women and men. It is important to note, however, that 22% of participants in the study came from a volunteer pool and some participants were also selected from the ARIC study which limits the generalizability of the study [25–28].

The current analysis aims to address some of the limitations in the existing literature by examining relationships between perceived neighborhood conditions, measured over time, and key cardiometabolic outcomes, within a randomly selected cohort living in two separate low-income, predominantly African American neighborhoods. Data from the study offers an opportunity to examine how changes in neighborhood conditions may be associated with cardiometabolic outcomes among a randomly selected cohort of predominantly African American, low-income residents. In this analysis, we attempt to determine how changes in participants’ perceptions of neighborhood environment with respect to infrastructure, safety, aesthetics, and satisfaction with one’s neighborhood as a place to live are associated with cardiometabolic outcomes. Further, given the known differences in cardiometabolic risk factors by sex [29] and that prior analyses in this cohort have shown sex differences health outcomes with neighborhood characteristics (i.e., walkability, crime and physical activity) [30], we hypothesized that the relationship between perceived neighborhood change and cardiometabolic outcomes would differ by sex.

## Methods

### Design overview

This analysis utilizes data from the Pittsburgh Hill/Homewood Research on Neighborhood Change and Health (PHRESH) Study, a series of projects which utilize a natural experiment to investigate how changes in neighborhood conditions influence health in a randomly selected cohort of residents in two similar, predominantly African American (> 90%), low-income neighborhoods in Pittsburgh, PA: the Hill District and Homewood [31]. One of the neighborhoods in this study (Hill District) has been undergoing substantial neighborhood revitalization investments, including the opening of a new full-service supermarket and other commercial development, renovation of green space and renovation and rebuilding of public housing, whereas the other neighborhood (Homewood), has been experiencing fewer neighborhood investments. The details of this study have been described elsewhere [31]. Briefly, participants were recruited in 2011 from a random sample of households drawn from a complete list of residential addresses generated by the Pittsburgh Neighborhood and Community Information System. Households were enrolled in-person by data collectors, who were recruited from the neighborhoods and trained to enroll households through door-to-door recruitment of the selected addresses.

### Study participants

The current study focuses on participants’ perceptions of changes in neighborhood conditions between 2013 and 2016 and cardiometabolic outcomes assessed in 2016 (the year cardiometabolic outcomes were added). A total of 710 participants were included in the cohort in both 2013 and 2016. Given that the primary aims of this manuscript are to examine the impact of perceived changes in existing neighborhood conditions on cardiometabolic outcomes, we further excluded participants who moved out of their original neighborhood between 2013 and 2016, and who did not have measured cardiometabolic outcomes, yielding a total sample of 622 participants. Compared to the overall PHRESH sample (710 participants), participants in the analysis sample were significantly older, had higher income, and lived in the neighborhood longer (all *p* < 0.05), which could attenuate the relationship between perceived neighborhood change and cardiometabolic outcomes.

Beginning with the 2016 data collection, as part of a new study focused on heart health, participants were asked to provide non-fasting blood samples for the measurement of cardiometabolic outcomes including hemoglobin A1c (HbA1c), and high-density lipoprotein cholesterol (HDL-c). As part of the household interview, participants’ blood pressure, weight and height were measured by the data collectors and body mass index (BMI) was calculated (kg/m^2^). Due to the fact that not all participants agreed to provide blood samples, there is variation in the sample sizes for each of the outcomes. However, to maximize sample sizes and avoid biasing results by dropping observations we elected to maintain the sample for each of the different outcomes. Overall, we calculated BMI from 620 participants, blood pressure from 583 participants, HbA1c values from 383 participants, and HDL-c values from 388 participants. We also examined whether the subsample who participated in the blood draw differs from the overall analytic sample. Those who participated in the blood draw had lived in their respective neighborhoods for a shorter duration of time compared to those who did not participate in the blood sample. There were no other differences between the subsample with blood draws and the overall analytic sample.

### Main predictor variables

We examined four subjective neighborhood characteristics over time: neighborhood infrastructure, neighborhood aesthetics, perceived safety, and neighborhood satisfaction (see questionnaire in Additional file 1). Perception of participants’ neighborhood was measured using subscales adapted from the Neighborhood Environment Walkability Scale (NEWS) [32] as well as neighborhood infrastructure and safety developed by Sampson et al. [33] While these four scales do represent different constructs they are mostly related to aspects of the built environment (as defined by Centers for Disease Control and Prevention, includes all of the physical parts of where we live and work (e.g., homes, buildings, streets, open spaces, and infrastructure), https://www.cdc.gov/nccdphp/dnpao/state-local-programs/built-environment-assessment/index.htm), however, several items in the safety scale and neighborhood satisfaction also address aspects of the social environment. In this analysis, we conceptualized that perceptions of built environment aspects of neighborhood and cardiometabolic health would be affected through health behaviors including, but not limited to, diet and physical activity.

#### Neighborhood infrastructure

Perceived infrastructure was obtained by averaging responses to five Likert scale items all with a range of one to five. The items included “there are sidewalks on most of the streets in your neighborhood”, “your neighborhood streets are well lit at night”, “people walking on the streets in your neighborhood can be easily seen by people in their homes”, “there are crosswalks/pedestrian signals to help people walking cross busy streets in your neighborhood”, and “the sidewalks in your neighborhood are well maintained, paved and don’t have cracks”. Higher scores indicated a better perceived infrastructure rating. Cronbach’s alpha for internal consistency as reported by Sampson et al. was α = 0.61 [33].

#### Neighborhood aesthetics

Perceived neighborhood aesthetics were obtained by averaging responses to three Likert scale items all with a range of one to five [32]. The items included “there are many interesting things to look at while walking in your neighborhood”, “there are many attractive natural sights in your neighborhood such as landscaping or views”, and “there are attractive buildings/homes in your neighborhood”. Higher scores for perceived aesthetics indicates greater perceived aesthetics.

#### Neighborhood safety

Perceived safety was obtained by averaging responses to four Likert scale items all with a range of one to five. The items included “you feel safe walking in your neighborhood during the day”, “you feel safe walking in your neighborhood during the evening”, “your neighborhood is safe from crime” and “violence is a problem in your neighborhood” the last of which was reverse coded. Higher scores for perceived safety indicated a better safety rating. Cronbach’s alpha for internal consistency as indicated by Sampson et al. was α = 0.85 [33].

#### Neighborhood satisfaction

Perceived satisfaction with one’s neighborhood as a place to live was measured using a single question [34] on a scale with responses which included, “very satisfied,” “satisfied,” “dissatisfied,” “very dissatisfied,” and “neutral.” Higher scores indicated higher neighborhood satisfaction. This measure has been used in previous longitudinal survey assessments of neighborhood conditions [34].

#### Changes in perceived neighborhood conditions

For each of the neighborhood conditions, we calculated change scores between 2016 and 2013 for each measure separately. For each measure, change in neighborhood perceptions were dichotomized to compare residents who perceived improvement in each of the neighborhood (change score ≥ 1) characteristics to those who perceived either no change or worsening of the neighborhood characteristics (change score < 1) based on a small proportion of participants indicating that they perceived a worsening in any of the neighborhood characteristics.

### Outcome measures

Two blood pressure measurements (taken 60 s apart) were obtained during an in-home assessment using a Micro Life automated blood pressure monitor after the participant had been seated for 5 min. The average of the two measurements was used to calculate the average systolic (SBP) and diastolic blood pressure (DBP). Interviewers measured height to the nearest eighth-inch using a carpenter’s square (triangle) and an eight-foot folding wooden ruler marked in inches. Interviewers measured weight to the nearest tenth-pound using a Seca Robusta 813 digital scale. BMI (kg/m2) was calculated from participants’ measured height and weight.

All other cardiometabolic indicators were measured via collection of non-fasting blood samples in the research clinic or in the participant’s home. Blood samples were obtained from the antecubital vein by a trained phlebotomist, while the participant was seated. Assays for HbA1c and HDL-c and were performed at the University of Pittsburgh Heinz Nutrition Laboratory at the Graduate School of Public Health. Both HbA1c and HDL-c outcomes were selected as the main outcomes for this study as they can be obtained with a non-fasting blood draw, which we determined was essential to reduce participant burden in this underrepresented sample.

### Covariates

Socio-demographics (age, education, income, marital status) were assessed by questionnaire. Race was assessed using the standard measure from government surveys which ask about ethnicity (Hispanic or Latino origin) and subsequently, race. Physical functioning was measured using a subscale of the SF-36 [35], which asks how much participants’ health limited their functioning during each of 10 activities (e.g., “doing moderate activities, such as moving a table, pushing a vacuum cleaner or “climbing one flight of stairs,”). Higher scores indicate better physical functioning. Participants were also asked by questionnaire how long they have lived in their current neighborhood. All covariates were assessed in 2013.

### Statistical methods

As described above, changes in each of the neighborhood perception scales (infrastructure, safety, aesthetics, and satisfaction) were determined by subtracting the 2016 Neighborhood Perception subscales from the corresponding 2013 subscales for each individual. Participants were then categorized into groups based on whether they perceived improvement, no change, or worsening in each of the perceived neighborhood characteristics over the time period. Due to known differences in the clinical manifestation of CVD between males and females, differences in participant characteristics and neighborhood perceptions were compared by sex using Chi-square and Kruskal-Wallis tests for categorical and continuous variables, respectively.

Multivariable linear regression was used to measure the associations between changes in each of the perceived neighborhood characteristics between 2013 and 2016 with cardiometabolic outcomes (BMI, BP, HbA1c, HDL-c) assessed in 2016. In each of these models, change in neighborhood perceptions were dichotomized to compare residents who perceived improvement in each of the neighborhood characteristics to those who perceived either no change or worsening of the neighborhood characteristics based on a small proportion of participants indicating that they perceived a worsening in any of the neighborhood characteristics. Models were adjusted for age, sex, education, household income, marital status, physical functioning as measured by the SF-36 scale, and years spent in neighborhood. Given that the original study sampled from two separate neighborhoods and employed a natural experiment design, an indicator variable for neighborhood was included in the models. In order to determine whether observed associations differed by sex, sex-stratified models were analyzed. Although we did not have blood pressure or blood measures collected before 2016, we do have baseline (2013) measures of BMI. Therefore, we conducted sensitivity analyses that additionally controlled for BMI to explore whether changes in neighborhood conditions predicted cardiometabolic outcomes, after adjustment for baseline BMI. All analyses were conducted using SAS 9.3 (SAS Institute, Cary, NC.) and *p*-values of < 0.05 were considered statistically significant.

## Results

### Sociodemographic characteristics, neighborhood variables, and Cardiometabolic outcomes

Characteristics of the study sample by sex are presented in Table 1. Participants were on average 58 years old. Most participants were high school graduates (41%) or had some college education (32%), and the median household income among the sample was $12,500. Few participants were married (18%). Most participants were long-term residents having lived in their respective neighborhoods on average 30 years. The median physical functioning score, as measured by the SF-36 scale, was 70. All baseline sociodemographic characteristics were similar by sex.

**Table 1:** Characteristics of the Study Sample by Sex

Characteristic	Total	Female (*n* = 494)	Male (*n* = 128)	*p*-value
Sociodemographics				
Age (years)	58.0 (48.-68.)	57 (47–69)	60 (51–66)	0.6334
Highest Education				0.4214
< HS	81 (13%)	65 (13%)	16 (13%)	
HS	257 (41%)	208 (42%)	49 (38%)	
Some college/Tech	200 (32%)	160 (32%)	40 (31%)	
College/Grad School	84 (14%)	61 (12%)	23 (18%)	
Married	111 (18%)	21 (16%)	90 (18%)	0.6332
Household Income	12,500 (7500-25,000)	12,500 (7500-25,000)	17,500 (7500-35,000)	0.1460
Physical Function	70.0 (45.0–90.0)	70 (45–90)	75 (50–95)	0.1478
Time Spent in Neighborhood (years)	31.0 (9.0–50.0)	31 (8–50)	30 (9–50)	0.8091
Baseline Neighborhood Scales				
Infrastructure Scale	3.2 (2.8–3.6)	3.2 (2.8–3.6)	3.2 (2.8–3.6)	0.7079
Perceived Safety Scale	3.0 (2.5–3.5)	3.0 (2.5–3.5)	3.0 (2.5–3.5)	0.4643
Aesthetics Scale	2.7 (2.0–4.0)	2.7 (2.0–3.7)	2.7 (2.0–4.0)	0.8849
Baseline Neighborhood Satisfaction	4.0 (3.0–4.0)	4.0 (3.0–4.0)	4.0 (3.0–4.0)	0.4538
Perceived Change in Neighborhood				
Improvement in Infrastructure	301 (48%)	238 (48%)	63 (49%)	0.8337
Improvement in Safety	293 (47%)	238 (48%)	55 (43%)	0.2927
Improvement in Aesthetics	283 (46%)	226 (46%)	57 (45%)	0.8052
Improvement in Neighborhood Satisfaction	176 (28%)	153 (31%)	23 (18%)	0.0036
Cardiometabolic Outcomes (2016)				
BMI (kg/m [2])	30.0 (25.4–35.2)	30.5 (26.0–35.7)	27.4 (23.1–32.4)	<.0001
SBP (mmHg)	127.0 (117.5–142.0)	126.0 (116.4–140.3)	131.0 (120.0–148.0)	0.0351
DBP (mmHg)	79.5 (71.5–87.0)	80.0 (71.4–87.0)	79.0 (72.0–87.0)	0.9404
HbA1c (%)	5.8 (5.5–6.3)	5.9 (5.5–6.3)	5.8 (5.4–6.2)	0.1490
HDL (mg/dL)	52.3 (43.2–61.5)	53.2 (43.4–63.5)	47.0 (41.7–57.0)	0.0052

Note: Results are presented as mean (range) for continuous variables and N (%) for categorical variables

Perceived neighborhood characteristics including infrastructure, safety, aesthetics, and satisfaction were similar by sex at baseline. When examining change in each neighborhood construct over time (2013–2016), many participants perceived improvements in infrastructure (48%), safety (47%) and aesthetics (46%) and these results were similar by sex (see Table 1). Overall, although 28% of participants perceived improvement in neighborhood satisfaction over time, more females (31%) reported improvements in neighborhood satisfaction than males (18%), *p* = .0036.

Consistent with prior research, cardiometabolic outcomes differed by sex. For instance, similar to national trends, females had higher BMI compared to males, mean 30.5 vs. 27.4 kg/m^2^, respectively, *p* < .0001. Females also had better SBP and HDL-c levels compared with males, and these differences were statistically significant (*p* = 0.0351 and *p* = 0.0052, respectively). Mean HbA1c values were similar for males and females at (5.8% vs. 5.9%; *p* = 0.1490).

### Changes in perceived neighborhood characteristics and Cardiometabolic outcomes

In the full sample, after adjustment for covariates, improvement in neighborhood safety was significantly associated with lower SBP, β = − 3.79 (*p* = 0.0361); see Table 2. Contrary to our hypothesis, however, perceived improvement in neighborhood safety was also associated with *higher* BMI levels, β =1.52 units higher, compared to those who perceived no improvement/worsening (*p* = 0.0162). After adjustment for baseline BMI (data not shown), the finding of improvement in neighborhood safety and lower SBP persisted (β = − 4.40), however, the relationship between improvement in neighborhood safety and higher BMI was no longer statistically significant. There were no other statistically significant associations between changes in neighborhood perceptions and cardiometabolic outcomes in the full sample. As the original study design was a natural experiment, we also ran models that included neighborhood as an interaction term and there were no significant findings (data not shown).

**Table 2:** Changes in Perceived Neighborhood Characteristics (2013–2016) and Cardiometabolic Outcomes

	BMI (*n* = 620)	SBP (*n* = 583)	DBP (*n* = 583)	HbA1c (*n* = 383)	HDL (*n* = 388)
Perceived Improvement in Infrastructure	0.72 (0.64)	−0.66 (1.81)	0.07 (0.97)	0.00 (0.12)	−1.01 (1.56)
Perceived Improvement in Aesthetics	1.17 (0.63)	0.23 (1.81)	− 0.01 (0.96)	0.05 (0.12)	−1.30 (1.53)
Perceived Improvement in Safety	1.52 (0.63)*	−3.79 (1.80)*	−1.36 (0.96)	0.23 (0.12)	−0.75 (1.54)
Perceived Improvement in Neighborhood Satisfaction	0.11 (0.70)	−1.15 (2.0)	−0.83 (1.06)	−0.25 (0.13)	− 0.46 (1.69)

Note. Beta coefficients and standard errors reported; *p*-value < 0.05 considered statistically significant (*bold)

Estimates are adjusted for neighborhood, age, sex, household income, education, marital status, physical function, and years spent in neighborhood

In follow-up models which stratified by sex (data not shown), improvements in neighborhood aesthetics and safety were significantly associated with higher BMI (β = 1.57, *p* = 0.0292, and β = 2.28, *p* = 0.0015, respectively) among females only. Additionally, among females perceived improvement in safety was significantly associated with higher HbA1c, % (β = 0.27, *p* = 0.0489). However, these relationships did not remain statistically significant after adjustment for baseline BMI (data not shown). No statistically significant associations were observed between perceived improvements in any of the neighborhood constructs with the measured health outcomes among males. Notably, the significant association between perceived safety and lower SBP observed among the entire sample did not persist in the stratified models; however, the relationship maintained a similar direction in both males and females.

## Discussion

Overall, participants perceived improvements in their neighborhood infrastructure, safety, aesthetics and to some extent, overall neighborhood satisfaction. Improvements in neighborhood safety between 2013 and 2016 were associated with higher BMI and lower SBP as measured in 2016. When the results were adjusted for BMI, only the relationship between improvements in perceived safety and lower SBP persisted. These results were based on a sample of predominantly African American residents followed in the same neighborhoods over time with measured cardiometabolic risk factors.

The findings from this analysis suggest that perceived neighborhood characteristics may have differing associations with multiple cardiometabolic outcomes (BMI, SBP). This highlights the complexity of the associations between neighborhood characteristics and health as well as the importance of considering how changes in perceived neighborhood characteristics associate with multiple cardiometabolic risk factors, and how associations may be sex dependent. The finding that improvements in neighborhood safety were associated with higher BMI and HbA1c levels in females were contrary to the direction that we hypothesized. In general, the cross-sectional literature shows a consistent relationship between neighborhood disadvantage and obesity/higher BMI levels, and cardiometabolic risk factors [36, 37]. For example, data from the Jackson Heart Study showed that neighborhood disadvantage was associated with a 25% increase of CVD and greater cumulative biological risk (using eight biomarkers of cardiovascular, metabolic, inflammatory, and neuroendocrine systems) [38–40]. Moreover, higher neighborhood-levels of violence and disorder were associated with 30% higher odds of smoking and lower neighborhood social cohesion was associated with higher odds of smoking and heavy alcohol use [41]. Thus, we hypothesized that increased neighborhood safety would be associated with lower cardiometabolic risk factors through better health behaviors such as smoking and lower BMI potentially through mechanisms such as increased physical activity. It is important to note that this unexpected finding did not persist after adjusting for baseline BMI.

We did not find any comparable studies in the literature to interpret this unexpected finding, however, other studies do show paradoxical relationships for BMI among African American populations, particularly women, compared to other populations [42, 43]. For example, significant interactions by race and poverty have been shown where African Americans who were living in poverty had lower BMI, waist circumference, and higher HDL cholesterol compared to those not living in poverty, whereas the opposite associations were shown for Whites [42]. In older adults, particularly those over age 65, moderate obesity later in life might improve survival and alleviate frailty [44]. Further, given the age of the sample, many of our participants were already diagnosed and were taking medication for risk factors such as high blood pressure and high cholesterol. This could have influenced our results, but the timing of the questions did not allow for us to adjust for this. We look forward to continuing to follow our cohort over time to better understand the predictors and trajectories of BMI and cardiometabolic health.

Despite its unique strengths, our study had several limitations including only one-time point for our assessment of cardiometabolic outcomes. This limits our ability to detect how changes in neighborhood environment affect these outcomes over time, however, we maximized data from multiple time-points for our exposure measures to predict cardiometabolic outcomes and adjusted for baseline BMI in sensitivity analyses. Another limitation is that in these analyses we excluded those PHRESH participants who moved out of the neighborhoods during our study. While we are still tracking those participants, we felt that it was crucial to assess change in neighborhood among those who were consistently living in those neighborhoods, as there may be other important mechanisms that account for associations between changes in neighborhood characteristics and cardiometabolic outcomes among movers (e.g., impact on social cohesion/ connectedness). Finally, we did have multiple comparisons which increases the chance of type 1 error, however, our main finding between perceived safety and systolic blood pressure was significant at the *p* < .01 level and not just *p* < 0.05. It is also important to note that there are many pathways that could link to neighborhood factors to cardiometabolic outcomes. Our study conceptualized and is attempting to assess two pathways: (1) the built or physical environment pathway affecting diet and physical activity and more recently, sleep, and (2) a social/stress pathway, referred to as the “social environment [45], which can have a direct (i.e., sympathetic nervous system, visceral fat accumulation) and indirect (i.e. unhealthy diet, sedentary behaviors, poor sleep) effect on obesity and cardiometabolic risk factors. This manuscript focused on the first pathway, however we acknowledge the other potential pathways and how they could be impacted by neighborhood socioeconomic status, racial segregation, and institutional racism.

## Conclusions

Studies of neighborhood factors and cardiometabolic health among predominantly African American populations are few, especially those that have longitudinal assessments of neighborhood conditions or outcomes. One study conducted an analysis of neighborhood-level socio-economic deprivation and changes in BMI within a multi-ethnic population within the Dallas Heart Study [46]. Results showed that living in more socioeconomically deprived neighborhoods was associated with weight gain in those participants who lived in those neighborhoods over a longer period of time. While these results are not directly comparable to our study as they did not present results for African Americans, specifically, and used census-based measures to assess neighborhood factors only at one time point, it does support the impact that neighborhood factors can have on weight over time. Our study is one of few population-based studies conducted in a predominantly African American population with longitudinal assessments of neighborhood over time and measured cardiometabolic outcomes at a single time-point. More studies are needed to create a body of literature that elucidates the complexities of the longitudinal associations between neighborhood factors and health outcomes in this population.

## Supplementary information

**Additional file 1.** PHRESH 2013 Questionnaire.

## Data Availability

De-identified data needed to replicate analyses reported in this paper will be made available upon request from the first author with an appropriate data use agreement and approval from RAND’s Human Subjects Protection Committee and the University of Pittsburgh IRB.

## Abbreviations

A1c: HbA1c
BMI: Body Mass Index
DBP: Diastolic Blood Pressure
HDL-c: High-Density Lipoprotein Cholesterol
SBP: Systolic Blood Pressure
