# Supplementary material for: Changes in perceptions of neighborhood environment and Cardiometabolic outcomes in two predominantly African American neighborhoods
Source: BMC Public Health. 2020 Jan 14;20:52. doi: 10.1186/s12889-019-8119-9 (PMC6961335; doi:10.1186/s12889-019-8119-9)
Supplement: Supplementary file 1 — Additional file 1. PHRESH 2013 Questionnaire. [file 12889_2019_8119_MOESM1_ESM.docx]

**PHRESH *Plus* ID:** ___________________

**INTERVIEWER ID:** ______________

**DATE OF INTERVIEW:** _______________________

**DATE ENTERED IN COMPUTER:** _________________

**LOCATION OF INTERVIEW**

- Their Home
- Hill House
- Operation Better Block
- Other Location [SPECIFY] _____________________________

**NEIGHBORHOOD**

- HILL DISTRICT
- HOMEWOOD OR LARIMER
- OTHER

**[ALL QUESTIONS ARE CHOOSE 1 RESPONSE UNLESS OTHERWISE INDICATED.]**

**[IF RESPONDENT LIVES IN A NEIGHBORHOOD THAT IS NOT THE HILL DISTRICT OR HOMEWOOD OR LARIMER, SAY “YOUR NEIGHBORHOOD” WHERE INDICATED. “NEIGHBORHOOD” IS WHATEVER IT MEANS TO THEM. NOTE SOME QUESTIONS ARE SKIPPED FOR THESE RESPONDENTS.]**

Thank you for agreeing to participate. Before we begin, I need to let you know that if there are other people in the house, they cannot participate or help you answer the interview questions. The questions must be answered by you only. Some of these questions are rather personal and I’d prefer to go through the questions with you alone. If someone else sits in on your interview, that will be your decision, however they must not offer to you with any of the questions. Do you have any questions before we begin?

# DOMAIN 1: PHYSICAL ACTIVITY/ACTIVE TRANSPORT

## Primary Physical Activity Location

We’d like to know where you have gone most often in the past month to engage in physical activity, either indoors or outdoors. For this question, please consider physical activity as something that you do for at least 10 minutes that takes at least moderate physical effort and makes you breathe somewhat harder than normal, including brisk walking.

**[FLIP TO CARD 1]**

1.1 In the past month, where have you gone most often for physical activity?

**[IF THERE ARE TWO PLACES THE RESPONDENT GOES TO ENGAGE IN PHYSICAL ACTIVITY EQUALLY, ASK RESPONDENT TO CHOOSE THE ONE PLACE THEY PREFER TO GO MOST OFTEN.]**

- Park (1)
- Trail or other walking/running path (2)
- Gym/recreation center (3)
- Exercise class (e.g., aerobics, Zumba) (4)
- Your own home (5)
- Home of a family member or a friend (6)
- Other (specify type of place) ___________________________________ (7)
- I don’t engage in physical activity. (8)
- DON’T KNOW (88)
- REFUSED (99)

**[TRY TO GET WHATEVER IDENTIFYING INFORMATION THE RESPONDENT CAN PROVIDE IF THEIR RESPONSE TO QUESTION 1.1 IS A PUBLIC PLACE. REFER TO CARDS 6 – 7 IF PLACE IS IN THE HILL DISTRICT OR HOMEWOOD OR LARIMER. THERE IS NO RESPONSE CARD FOR PLACES IN OTHER NEIGHBORHOODS.]**

Name of place: _____________________________________________

Address/cross streets of place: ___________________________________________

1.1a Is this place inside **[INSERT HILL DISTRICT/HOMEWOOD OR LARIMER/YOUR NEIGHBORHOOD]** or outside? [DO NOT READ THE FOLLOWING FOR RESIDENTS OF OTHER NEIGHBORHOODS:] For these questions, the Hill includes Upper Hill, Middle Hill, Crawford Roberts, Bedford Dwellings, Terrace Village, and Oak Hill. Homewood or Larimer includes Homewood West, Homewood North, Homewood South, and Larimer.

- Inside Hill/Homewood or Larimer/your neighborhood (1)
- Outside Hill/Homewood or Larimer/your neighborhood (2)
- DON’T KNOW (88)
- REFUSED (99)

## Walking and Use of Stairwells in Neighborhood

We’d like to know how often you walked to places in **[INSERT HILL DISTRICT/HOMEWOOD OR LARIMER/YOUR NEIGHBORHOOD]** for exercise, pleasure, or to get somewhere you needed to go in the past month.

**[FLIP TO CARD 2]**

1.2 In the past month, how often did you walk to places in **[INSERT HILL DISTRICT/HOMEWOOD OR LARIMER/YOUR NEIGHBORHOOD]** for exercise, pleasure, or to get somewhere you needed to go?

- At least once a day in the past month (1)
- 3-6 times a week in the past month (2)
- Once or twice a week in the past month (3)
- 2-3 times in the past month (4)
- Once in the past month (5)
- Never in the past month (6)
- DON’T KNOW (88)
- REFUSED (99)

**[SKIP 1.3a and 1.3b FOR RESIDENTS OF OTHER NEIGHBORHOODS.]**

We’d also like to know about your use of the city steps and outdoor stairwells in **[INSERT HILL DISTRICT /HOMEWOOD OR LARIMER]** for exercise, pleasure, or to get somewhere you needed to go in the past month.

1.3a Are you aware that there are city steps and outdoor stairwells in **[INSERT HILL DISTRICT/HOMEWOOD OR LARIMER]**?

- YES (1)🡪**GO to 1.3b**
- NO (2)🡪**SKIP 1.3b and GO TO 1.4**
- DON’T KNOW (88)🡪**SKIP 1.3b and GO TO 1.4**
- REFUSED (99)🡪**SKIP 1.3b and GO TO 1.4**

1.3b In the past month, how often did you use the city steps and outdoor stairwells in **[INSERT HILL DISTRICT /HOMEWOOD OR LARIMER]** for exercise, pleasure, or to get somewhere you need to go?

- At least once a day in the past month (1)
- 3-6 times a week in the past month (2)
- Once or twice a week in the past month (3)
- 2-3 times in the past month (4)
- Once in the past month (5)
- Never in the past month (6)
- DON’T KNOW (88)
- REFUSED (99)

## Frequency of Physical Activity

We are interested in finding out about the kinds of physical activities that residents do as part of their everyday lives. The questions will ask you about the time you spent being physically active in the last 7 days. Please answer each question even if you do not consider yourself to be an active person. Please think about the activities you do at work, as part of your house and yard work, to get from place to place, and in your spare time for recreation, exercise or sport.

Think about all the vigorous activities that you did in the last 7 days. Vigorous physical activities refer to activities that take strenuous physical effort and make you breathe much harder than normal. Think only about those physical activities that you did for at least 10 minutes at a time. **[REPEAT THIS DEFINITION OF VIGOROUS PHYSICAL ACTIVITY AS NEEDED FOR THE FOLLOWING QUESTIONS.]**

1.4 During the last 7 days, on how many days did you do vigorous physical activities like heavy lifting, digging, aerobics, Zumba, or fast bicycling?

_____ days per week

- No vigorous physical activities (0) 🡪 **SKIP 1.5 and GO TO** **1.6**
- DON’T KNOW (88)
- REFUSED (99)

1.5 How much time did you usually spend doing vigorous physical activities on one of those days?

_____ hours per day

_____ minutes per day

- DON’T KNOW (88)
- REFUSED (99)

Think about all the moderate activities that you did in the last 7 days. Moderate activities refer to activities that take moderate physical effort and make you breathe somewhat harder than normal. Think only about those physical activities that you did for at least 10 minutes at a time. **[REPEAT THIS DEFINITION OF MODERATE PHYSICAL ACTIVITY AS NEEDED FOR THE FOLLOWING QUESTIONS.]**

1.6 During the last 7 days, on how many days did you do moderate physical activities like carrying light loads, bicycling at a regular pace, doubles tennis, or exercise and dance classes? Do not include walking.

_____ days per week

- No moderate physical activities (0) 🡪 **SKIP 1.7 and GO TO** **1.8**
- DON’T KNOW (88)
- REFUSED (99)

1.7 How much time did you usually spend doing moderate physical activities on one of those days?

_____ hours per day

_____ minutes per day

- DON’T KNOW (88)
- REFUSED (99)

Think about the time you spent walking in the last 7 days. This includes at work and at home, walking to travel from place to place, and any other walking that you have done solely for recreation, sport, exercise, or leisure.

1.8 During the last 7 days, on how many days did you walk for at least 10 minutes at a time?

_____ days per week

- No walking (0) 🡪 **SKIP 1.9 and GO TO** **1.10a**
- DON’T KNOW (88)
- REFUSED (99)

1.9 How much time did you usually spend walking on one of those days?

_____ hours per day

_____ minutes per day

- DON’T KNOW (88)
- REFUSED (99)

**[FLIP TO CARD 3]**

***Active Transport***

Now think only about the walking you might have done to travel to and from work, to do errands, or to go from place to place; do not include walking that you have done solely for recreation, sport, exercise, or leisure.

1.10a During the last 7 days, on how many days did you walk for at least 10 minutes at a time to go from place to place?

- 0 days (0) 🡪 **SKIP 1.10b and GO TO** **1.11**
- 1 day (1)
- 2 days (2)
- 3 days (3)
- 4 days (4)
- 5 days (5)
- 6 days (6)
- 7 days (7)
- DON’T KNOW (88)
- REFUSED (99)

1.10b How much time did you usually spend on one of those days walking from place to place?

______ hours per day

______ minutes per day

- DON’T KNOW (88)
- REFUSED (99)

This next question is about the time you spent sitting on weekdays during the last 7 days. Include time spent at work, at home, while doing course work and during leisure time. This may include time spent sitting at a desk, visiting friends, reading, or sitting or lying down to watch television.

1.11 During the last 7 days, how much time did you spend sitting on a week day?

_____ hours per day

_____ minutes per day

- DON’T KNOW (88)
- REFUSED (99)

## Screen Time

The following questions ask about your use of different media. In the next two questions, we ask about “using electronic media.” By using electronic media we mean watching TV, movies, or videos; visiting Facebook, Twitter, YouTube, or other social networking sites; visiting other websites; playing online games, computer games, or other videogames; texting; instant messaging; online chatting; emailing; and using apps.

**[FLIP TO CARD 4]**

1.12 On a typical day, how many hours do you spend using electronic media outside of work?

- Less than 1 hour (1)
- 1 to less than 2 hours (2)
- 2 to less than 3 hours (3)
- 3 to less than 4 hours (4)
- 4 to less than 5 hours (5)
- 5 to less than 6 hours (6)
- 6 to less than 7 hours (7)
- 7 to less than 8 hours (8)
- 8 to less than 9 hours (9)
- 9 to less than 10 hours (10)
- 10 hours or more (11)
- DON’T KNOW (88)
- REFUSED (99)

**[FLIP TO CARD 5]**

1.13 On a typical day, which do you spend the most time doing?

- Watching TV, movies, or videos (1)
- Visiting Facebook, Twitter, YouTube, or other social networking sites (2)
- Playing online games, computer games, other video games, or apps (3)
- Texting, instant messaging, online chatting, or emailing (4)
- Surfing the Internet (5)
- Other (specify): ________________________________ (6)
- DON’T KNOW (88)
- REFUSED (99)

# DOMAIN 2: NEIGHBORHOOD PARK/PLAYGROUND USE

**[SKIP QUESTIONS 2.1 THROUGH 2.4c FOR RESIDENTS OF OTHER NEIGHBORHOODS.]**

The next several questions are about your use of parks and playgrounds in your neighborhood, that is, the **[INSERT HILL DISTRICT/HOMEWOOD OR LARIMER]**. (For these questions, the Hill includes Upper Hill, Middle Hill, Crawford Roberts, Bedford Dwellings, Terrace Village, and Oak Hill. Homewood or Larimer includes Homewood West, Homewood North, Homewood South, and Larimer.)

2.1 Have you ever visited the **[INSERT NAME OF PARK OR PLAYGROUND FROM LIST BELOW]**?

| **[HILL DISTRICT – FLIP TO CARD 6]** | **Yes** | **No** | **DON’T KNOW** | **REFUSED** |
| --- | --- | --- | --- | --- |
| 1. Al “Turk” Graham Park | (1) | (5) | (88) | (99) |
| 1. Ammon Recreational Center (aka Ammons) | (1) | (5) | (88) | (99) |
| 1. The Basketball court in the Bentley Drive housing complex | (1) | (5) | (88) | (99) |
| 1. Cliffside Parklet | (1) | (5) | (88) | (99) |
| 1. Dunseith Street Playground (aka Shalane’s Playground) | (1) | (5) | (88) | (99) |
| 1. Granville Parklet (aka Ozanam Park) | (1) | (5) | (88) | (99) |
| 1. Herron Hill tennis courts (aka Alfred B. Carl Courts) | (1) | (5) | (88) | (99) |
| 1. Kennard Field | (1) | (5) | (88) | (99) |
| 1. Martin Luther King Field | (1) | (5) | (88) | (99) |
| 1. Robert E. (Pappy) Williams Park on Sugar Top | (1) | (5) | (88) | (99) |
| 1. Schenley Ball Field | (1) | (5) | (88) | (99) |
| 1. Vincennes Park (behind old firehouse apartments) | (1) | (5) | (88) | (99) |
| 1. West Penn Recreational Center (aka West Penn) | (1) | (5) | (88) | (99) |

**[IF RESPONDENT ANSWERS “NO” FOR ALL ITEMS (2.1a TO 2.1m) ABOVE, GO TO QUESTION 2.9.**]

| **[HOMEWOOD OR LARIMER - FLIP TO CARD 7]** | **Yes** | **No** | **DON’T KNOW** | **REFUSED** |
| --- | --- | --- | --- | --- |
| aa. Baxter Park | (1) | (5) | (88) | (99) |
| bb. Chadwick Playground | (1) | (5) | (88) | (99) |
| cc. Dallas Playground (aka North Dallas Park) | (1) | (5) | (88) | (99) |
| dd. Faison Playground | (1) | (5) | (88) | (99) |
| ee. Homewood North Park | (1) | (5) | (88) | (99) |
| ff. Homewood Playground (aka Willie Stargell Field) | (1) | (5) | (88) | (99) |
| gg. June Bug / YMCA Basketball Court | (1) | (5) | (88) | (99) |
| hh. KaBoom Playground (aka Tioga Playground) | (1) | (5) | (88) | (99) |
| ii. Westinghouse Park | (1) | (5) | (88) | (99) |
| jj. Wilkinsburg Playground | (1) | (5) | (88) | (99) |
| kk. YMCA Basketball Court | (1) | (5) | (88) | (99) |
| ll. Highland Park | (1) | (5) | (88) | (99) |
| mm. Mellon Park | (1) | (5) | (88) | (99) |
| nn. Larimer Playground | (1) | (5) | (88) | (99) |
| oo. Frick Park | (1) | (5) | (88) | (99) |
| pp. East Liberty Park/Ballfield | (1) | (5) | (88) | (99) |

**[IF RESPONDENT ANSWERS “NO” FOR ALL ITEMS (2.1aa TO 2.1pp) ABOVE, GO TO QUESTION 2.9.**]

**[FLIP TO CARD 8]**

Now I’m going to ask you some questions about each of the playgrounds and parks you have visited. **[ASK QUESTION 2.2 ONLY FOR PARK/PLAYGROUND(S) THAT RESPONDENT SAID “YES” THEY HAVE VISITED IN QUESTION 2.1]**

2.2 In the past month, how often did you visit **[INSERT NAME OF APPROPRIATE PARK OR PLAYGROUND FROM LIST BELOW]**?

| **HILL DISTRICT** | **At least once a day** | **3 – 6X a week** | **1 – 2X  a week** | **2 – 3 times a month** | **Once a month** | **Never** | **DON’T KNOW** | **REFUSED** |
| --- | --- | --- | --- | --- | --- | --- | --- | --- |
| 1. Al “Turk” Graham Park | (1) | (2) | (3) | (4) | (5) | (0) | (88) | (99) |
| 1. Ammon Recreational Center (aka Ammons) | (1) | (2) | (3) | (4) | (5) | (0) | (88) | (99) |
| 1. Basketball court in the Bentley Drive housing complex | (1) | (2) | (3) | (4) | (5) | (0) | (88) | (99) |
| 1. Cliffside Parklet | (1) | (2) | (3) | (4) | (5) | (0) | (88) | (99) |
| 1. Dunseith Street Playground (aka Shalane’s Playground) | (1) | (2) | (3) | (4) | (5) | (0) | (88) | (99) |
| 1. Granville Parklet (aka Ozanam Park) | (1) | (2) | (3) | (4) | (5) | (0) | (88) | (99) |
| 1. Herron Hill tennis courts (aka Alfred B. Carl Courts) | (1) | (2) | (3) | (4) | (5) | (0) | (88) | (99) |
| 1. Kennard Field | (1) | (2) | (3) | (4) | (5) | (0) | (88) | (99) |
| 1. Martin Luther King Field | (1) | (2) | (3) | (4) | (5) | (0) | (88) | (99) |
| 1. Robert E. (Pappy) Williams Park on Sugar Top | (1) | (2) | (3) | (4) | (5) | (0) | (88) | (99) |
| 1. Schenley Ball Field | (1) | (2) | (3) | (4) | (5) | (0) | (88) | (99) |
| 1. Vincennes Park (behind old firehouse apartments) | (1) | (2) | (3) | (4) | (5) | (0) | (88) | (99) |
| 1. West Penn Recreational Center (aka West Penn) | (1) | (2) | (3) | (4) | (5) | (0) | (88) | (99) |

| **HOMEWOOD OR LARIMER** | **At least once a day** | **3 – 6X a week** | **1 – 2X  a week** | **2 – 3 times a month** | **Once a month** | **Never** | **DON’T KNOW** | **REFUSED** |
| --- | --- | --- | --- | --- | --- | --- | --- | --- |
| aa. Baxter Park | (1) | (2) | (3) | (4) | (5) | (0) | (88) | (99) |
| bb. Chadwick Playground | (1) | (2) | (3) | (4) | (5) | (0) | (88) | (99) |
| cc. Dallas Playground  (aka North Dallas Park) | (1) | (2) | (3) | (4) | (5) | (0) | (88) | (99) |
| dd. Faison Playground | (1) | (2) | (3) | (4) | (5) | (0) | (88) | (99) |
| ee. Homewood North Park | (1) | (2) | (3) | (4) | (5) | (0) | (88) | (99) |
| ff. Homewood Playground  (aka Willie Stargell Field) | (1) | (2) | (3) | (4) | (5) | (0) | (88) | (99) |
| gg. June Bug / YMCA Basketball Court | (1) | (2) | (3) | (4) | (5) | (0) | (88) | (99) |
| hh. KaBoom Playground  (aka Tioga Playground) | (1) | (2) | (3) | (4) | (5) | (0) | (88) | (99) |
| ii. Westinghouse Park | (1) | (2) | (3) | (4) | (5) | (0) | (88) | (99) |
| jj. Wilkinsburg Playground | (1) | (2) | (3) | (4) | (5) | (0) | (88) | (99) |
| kk. YMCA Basketball Court | (1) | (2) | (3) | (4) | (5) | (0) | (88) | (99) |
| ll. Highland Park | (1) | (2) | (3) | (4) | (5) | (0) | (88) | (99) |
| mm. Mellon Park | (1) | (2) | (3) | (4) | (5) | (0) | (88) | (99) |
| nn. Larimer Playground | (1) | (2) | (3) | (4) | (5) | (0) | (88) | (99) |
| oo. Frick Park | (1) | (2) | (3) | (4) | (5) | (0) | (88) | (99) |
| pp. East Liberty Park/Ballfield | (1) | (2) | (3) | (4) | (5) | (0) | (88) | (99) |

**[IF THE RESPONDENT’S HIGHEST FREQUENCY OF VISITATION APPLIES TO MORE THAN THREE PARKS (FOR EXAMPLE, RESPONDENT SAYS HE/SHE VISITED FOUR PARKS 1-2 TIMES A WEEK, ASK QUESTION 2.2a TO NARROW IT DOWN TO THE THREE PARKS THAT HE/SHE VISITED THE MOST FREQUENTLY IN THE PAST MONTH. IF THE RESPONDENT MOST FREQUENTLY VISITED ONLY THREE OR FEWER PARKS, SKIP 2.2a AND GO TO QUESTION 2.3a.]**

2.2a You said that you visited **[INSERT NAMES OF THE FOUR OR MORE PARKS VISITED AT THE RESPONDENT’S HIGHEST FREQUENCY, E.G., “ONE OR TWO TIMES A WEEK”]**. Which three of these parks did you visit most often in the past month?

**[HILL DISTRICT – FLIP TO CARD 6]**

**[HOMEWOOD OR LARIMER – FLIP TO CARD 7]**

Park Name #1: _______________________________

Park Name #2: _______________________________

Park Name #3: _______________________________

**[FROM RESPONSES GIVEN TO 2.2 OR 2.2a, ASK QUESTIONS 2.3 AND 2.4 ONLY ABOUT THE THREE PARKS THAT RESPONDENT SAID THEY VISITED MOST FREQUENTLY IN THE PAST MONTH.]**

For this question, please consider physical activity something that you did for at least 10 minutes that took at least moderate physical effort and made you breathe at least somewhat harder than normal, including brisk walking. **[ASK THIS QUESTION FOR EACH OF THE TOP 3 MOST FREQUENTLY VISITED PARK/PLAYGROUND(S).]**

2.3a On a typical day when you visited **[INSERT NAME OF FIRST PARK]**, did you engage in physical activity for at least 10 minutes?

- YES (1)
- NO (5)🡪**SKIP 2.4a and GO TO 2.5a**
- DON’T KNOW (88)🡪**SKIP 2.4a and GO TO 2.5a**
- REFUSED (99) 🡪**SKIP 2.4a and GO TO 2.5a**

2.3b On a typical day when you visited **[INSERT NAME OF SECOND PARK]**, did you engage in physical activity for at least 10 minutes?

- YES (1)
- NO (5)🡪**SKIP 2.4b and GO TO 2.5a**
- DON’T KNOW (88)🡪**SKIP 2.4b and GO TO 2.5a**
- REFUSED (99) 🡪**SKIP 2.4b and GO TO 2.5a**

2.3c On a typical day when you visited **[INSERT NAME OF THIRD PARK]**, did you engage in physical activity for at least 10 minutes?

- YES (1)
- NO (5)🡪**SKIP 2.4c and GO TO 2.5a**
- DON’T KNOW (88)🡪**SKIP 2.4c and GO TO 2.5a**
- REFUSED (99) 🡪**SKIP 2.4c and GO TO 2.5a**

**[FOR EACH PARK IN 2.3a-c TO WHICH THE ANSWER WAS “YES,” ASK 2.4a-c. IF THE ANSWER WAS “NO” FOR ALL PARKS IN 2.3a-c, GO TO 2.5a.]**

**[FLIP TO CARD 9]**

2.4a. How much time did you spend engaging in at least moderate physical activity when you were at **[INSERT NAME OF FIRST PARK]**?

- 10-19 minutes (1)
- 20-29 minutes (2)
- 30-39 minutes (3)
- 40-49 minutes (4)
- 50-59 minutes (5)
- An hour or more (6)
- DON’T KNOW (88)
- REFUSED (99)

2.4b. How much time did you spend engaging in at least moderate physical activity when you were at **[INSERT NAME OF SECOND PARK]**?

- 10-19 minutes (1)
- 20-29 minutes (2)
- 30-39 minutes (3)
- 40-49 minutes (4)
- 50-59 minutes (5)
- An hour or more (6)
- DON’T KNOW (88)
- REFUSED (99)

2.4c. How much time did you spend engaging in at least moderate physical activity when you were at **[INSERT NAME OF THIRD PARK]**?

- 10-19 minutes (1)
- 20-29 minutes (2)
- 30-39 minutes (3)
- 40-49 minutes (4)
- 50-59 minutes (5)
- An hour or more (6)
- DON’T KNOW (88)
- REFUSED (99)

**[HILL DISTRICT – FLIP TO CARD 6]**

**[HOMEWOOD OR LARIMER – FLIP TO CARD 7]**

2.5a Which neighborhood park is closest to your home?

**[IF THE PARK CLOSEST TO THE RESPONDENT’S HOME ISN’T ON THE LIST, WRITE DOWN THE NAME AND ITS LOCATION AS OTHER. THERE IS NO LIST FOR RESIDENTS OF OTHER NEIGHBORHOODS, JUST WRITE IN THEIR RESPONSE.]**

Name of closest park (from list): ________________________________

Other, specify: ______________________________________________

**[FLIP TO CARD 10]**

2.5b Whether or not you’ve ever visited **[INSERT NAME OF PARK LOCATED CLOSEST TO RESPONDENT’S HOME]**, how safe do you think it is?

- Very safe (1) **🡪 SKIP 2.6 and GO TO 2.7**
- Safe (2) 🡪 **SKIP 2.6 and GO TO 2.7**
- Unsafe (3)
- Very unsafe (4)
- DON’T KNOW (88) 🡪 **SKIP 2.6 and GO TO 2.7**
- REFUSED (99) 🡪 **SKIP 2.6 and GO TO 2.7**

**[FLIP TO CARD 11]**

2.6 You said you think **[INSERT NAME OF PARK LOCATED CLOSEST TO RESPONDENT’S HOME]** is not safe. Why do you think it is not safe? **[CHOOSE ALL THAT APPLY]**

- Too crowded (1)
- People loitering (2)
- Too secluded (3)
- Crime or violence (4)
- People using or selling drugs (5)
- Poor lighting (6)
- Poor maintenance of park (7)
- Other, specify: _________________________________ (8)
- DON’T KNOW (88)
- REFUSED (99)

We would like to know how the playground/parks in **[INSERT HILL DISTRICT/HOMEWOOD OR LARIMER/YOUR NEIGHBORHOOD]** could be improved.

**[FLIP TO CARD 12]**

**[THIS QUESTION ASKS ABOUT NEIGHBORHOOD PARKS/PLAYGROUNDS IN GENERAL, AND IS NOT SPECIFIC TO THE PARKS/PLAYGROUNDS THE RESPONDENT HAS VISITED.]**

2.7 Whether or not you have visited the playgrounds or parks in **[INSERT HILL DISTRICT/ HOMEWOOD OR LARIMER/YOUR NEIGHBORHOOD]**, what additional activities, programs, facilities or amenities would you like to see in your neighborhood playground/parks that would make you more physically active?
**[CHOOSE ALL THAT APPLY]**

- Bicycle paths (1)
- Environment activities (2)
- Walking paths or trails (3)
- Lighting (4)
- Adult sports leagues (5)
- Youth sports leagues (6)
- Arts activities (7)
- Adult dance classes (8)
- Quiet spaces (9)
- Fitness classes (outdoor) (10)
- Playground equipment (11)
- Organized adventure walks (12)
- Park events/fairs (13)
- Park concerts/dances (14)
- More trees/landscaping (15)
- Garden area (16)
- Other, specify: __________________________________ (17)
- None of the above (18)
- DON’T KNOW (88)
- REFUSED (99)

**[FLIP TO CARD 13]**

## Perceived Accessibility of Neighborhood Parks and Playgrounds

Please tell me whether you strongly agree, agree, disagree, strongly disagree or neither agree or disagree with the following statement:

2.8 The playgrounds and parks in **[INSERT HILL DISTRICT/HOMEWOOD OR LARIMER/YOUR NEIGHBORHOOD]** are difficult to get to.

- Strongly agree (5)
- Agree (4)
- Disagree (2)
- Strongly disagree (1)
- Neither agree nor disagree (3)
- DON’T KNOW (88)
- REFUSED (99)

**[SKIP 2.9 FOR RESIDENTS OF OTHER NEIGHBORHOODS.]**

## Awareness of Neighborhood/Greenspace Renovations

2.9 Are you aware of the renovations to parks, playgrounds, city steps and outdoor stairwells, and walking paths in **[INSERT HILL DISTRICT/HOMEWOOD OR LARIMER]** that are scheduled to take place over the next few years?

- Yes (1)
- No (5)
- DON’T KNOW (88)
- REFUSED (99)

# domain 3: NEIGHBORHOOD PERCEPTIONS

The next several questions are about what you think of your neighborhood. **[DO NOT READ THE FOLLOWING FOR RESIDENTS OF OTHER NEIGHBORHOODS:]** For these questions, “neighborhood” is defined in the following way: **[FOR RESIDENTS OF THE HILL DISTRICT:]** Neighborhood includes Upper Hill, Middle Hill, Crawford Roberts, Bedford Dwellings, Terrace Village, and Oak Hill. **[FOR RESIDENTS OF HOMEWOOD OR LARIMER:]** Neighborhood includes Homewood West, Homewood North, Homewood South, and Larimer.

## Neighborhood Infrastructure for Physical Activity

For each of the following statements, would you say that you strongly agree, agree, disagree, strongly disagree, or are neutral — neither agree nor disagree?

*Streets and Sidewalks*

3.1 There are sidewalks on most of the streets in your neighborhood.

- Strongly agree (5)
- Agree (4)
- Disagree (2)
- Strongly disagree (1)
- Neither agree nor disagree (3)
- DON’T KNOW (88)
- REFUSED (99)

3.2 There is a grass or dirt strip that separates the streets from the sidewalks in your neighborhood.

- Strongly agree (5)
- Agree (4)
- Disagree (2)
- Strongly disagree (1)
- Neither agree nor disagree (3)
- DON’T KNOW (88)
- REFUSED (99)

3.3 Your neighborhood streets are well lit at night.

- Strongly agree (5)
- Agree (4)
- Disagree (2)
- Strongly disagree (1)
- Neither agree nor disagree (3)
- DON’T KNOW (88)
- REFUSED (99)

3.4 People walking on the streets in your neighborhood can be easily seen by people in their homes.

- Strongly agree (5)
- Agree (4)
- Disagree (2)
- Strongly disagree (1)
- Neither agree nor disagree (3)
- DON’T KNOW (88)
- REFUSED (99)

3.5 There are crosswalks and pedestrian signals to help people walking cross busy streets in your neighborhood.

- Strongly agree (5)
- Agree (4)
- Disagree (2)
- Strongly disagree (1)
- Neither agree nor disagree (3)
- DON’T KNOW (88)
- REFUSED (99)

3.6 The sidewalks in your neighborhood are well maintained: They are paved and don’t have a lot of cracks.

- Strongly agree (5)
- Agree (4)
- Disagree (2)
- Strongly disagree (1)
- Neither agree nor disagree (3)
- DON’T KNOW (88)
- REFUSED (99)

3.7 The streets in your neighborhood are hilly, making your neighborhood difficult to walk in.

- Strongly agree (5)
- Agree (4)
- Disagree (2)
- Strongly disagree (1)
- Neither agree nor disagree (3)
- DON’T KNOW (88)
- REFUSED (99)

**[SKIP QUESTIONS 3.8 THROUGH 3.10 FOR RESIDENTS OF OTHER NEIGHBORHOODS.]**

*Stairwells*

3.8 The city steps and outdoor stairwells in your neighborhood are well maintained.

- Strongly agree (5)
- Agree (4)
- Disagree (2)
- Strongly disagree (1)
- Neither agree nor disagree (3)
- DON’T KNOW (88)
- REFUSED (99)

3.9 The city steps and outdoor stairwells in your neighborhood are well lit.

- Strongly agree (5)
- Agree (4)
- Disagree (2)
- Strongly disagree (1)
- Neither agree nor disagree (3)
- DON’T KNOW (88)
- REFUSED (99)

3.10 The city steps and outdoor stairwells in your neighborhood are safe.

- Strongly agree (5)
- Agree (4)
- Disagree (2)
- Strongly disagree (1)
- Neither agree nor disagree (3)
- DON’T KNOW (88)
- REFUSED (99)

## Traffic Hazards

3.11 There is so much traffic along nearby streets that it makes it difficult or unpleasant to walk in your neighborhood.

- Strongly agree (5)
- Agree (4)
- Disagree (2)
- Strongly disagree (1)
- Neither agree nor disagree (3)
- DON’T KNOW (88)
- REFUSED (99)

3.12 The speed of traffic on most nearby streets is usually slow (30 mph or less).

- Strongly agree (5)
- Agree (4)
- Disagree (2)
- Strongly disagree (1)
- Neither agree nor disagree (3)
- DON’T KNOW (88)
- REFUSED (99)

3.13 Most drivers exceed the posted speed limits while driving in your neighborhood.

- Strongly agree (5)
- Agree (4)
- Disagree (2)
- Strongly disagree (1)
- Neither agree nor disagree (3)
- DON’T KNOW (88)
- REFUSED (99)

## Access to Services

3.14 Stores are within easy walking distance of your home.

- Strongly agree (5)
- Agree (4)
- Disagree (2)
- Strongly disagree (1)
- Neither agree nor disagree (3)
- DON’T KNOW (88)
- REFUSED (99)

3.15 There are many places to go within easy walking distance of your home.

- Strongly agree (5)
- Agree (4)
- Disagree (2)
- Strongly disagree (1)
- Neither agree nor disagree (3)
- DON’T KNOW (88)
- REFUSED (99)

3.16 It is easy to walk to a bus stop from your home.

- Strongly agree (5)
- Agree (4)
- Disagree (2)
- Strongly disagree (1)
- Neither agree nor disagree (3)
- DON’T KNOW (88)
- REFUSED (99)

Next, I have some more questions about your neighborhood.

## Neighborhood Satisfaction

**[FLIP TO CARD 14]**

3.17 All things considered, would you say you are very satisfied, satisfied, dissatisfied, very dissatisfied or neutral — neither satisfied nor dissatisfied with your neighborhood as a place to live?

- Very Satisfied (5)
- Satisfied (4)
- Dissatisfied (2)
- Very Dissatisfied (1)
- Neutral – neither satisfied nor dissatisfied (3)
- DON’T KNOW (88)
- REFUSED (99)

## Social Cohesion

**[FLIP TO CARD 13]**

For each of the following statements, would you say that you strongly agree, agree, disagree, strongly disagree, or are neutral - neither agree nor disagree?

3.18 People in this neighborhood are willing to help their neighbors.

- Strongly agree (5)
- Agree (4)
- Disagree (2)
- Strongly disagree (1)
- Neither agree nor disagree (3)
- DON’T KNOW (88)
- REFUSED (99)

3.19 This is a close-knit neighborhood.

- Strongly agree (5)
- Agree (4)
- Disagree (2)
- Strongly disagree (1)
- Neither agree nor disagree (3)
- DON’T KNOW (88)
- REFUSED (99)

3.20 People in this neighborhood can be trusted.

- Strongly agree (5)
- Agree (4)
- Disagree (2)
- Strongly disagree (1)
- Neither agree nor disagree (3)
- DON’T KNOW (88)
- REFUSED (99)

3.21 People in this neighborhood generally don’t get along with each other.

- Strongly agree (5)
- Agree (4)
- Disagree (2)
- Strongly disagree (1)
- Neither agree nor disagree (3)
- DON’T KNOW (88)
- REFUSED (99)

3.22 People in this neighborhood do not share the same values.

- Strongly agree (5)
- Agree (4)
- Disagree (2)
- Strongly disagree (1)
- Neither agree nor disagree (3)
- DON’T KNOW (88)
- REFUSED (99)

3.23 People in this neighborhood look out for one another, whether they are standing on the streets, sitting on their porches, or just walking around.

- Strongly agree (5)
- Agree (4)
- Disagree (2)
- Strongly disagree (1)
- Neither agree nor disagree (3)
- DON’T KNOW (88)
- REFUSED (99)

## Neighborhood Safety

**[DO NOT READ THESE INSTRUCTIONS FOR RESIDENTS OF OTHER NEIGHBORHOODS. If respondent needs a REMINDER, For these questions, “neighborhood” IS DEFINED IN THE FOLLOWING WAY: FOR RESIDENTS OF THE HILL DISTRICT, NEIGHBORHOOD INCLUDES UPPER HILL, MIDDLE HILL, CRAWFORD ROBERTS, BEDFORD DWELLINGS, TERRACE VILLAGE, AND OAK HILL**. **FOR RESIDENTS OF HOMEWOOD OR LARIMER, NEIGHBORHOOD INCLUDES HOMEWOOD WEST, HOMEWOOD NORTH, HOMEWOOD SOUTH, AND LARIMER.]**

3.24 You feel safe walking in your neighborhood during the day.

- Strongly agree (5)
- Agree (4)
- Disagree (2)
- Strongly disagree (1)
- Neither agree nor disagree (3)
- DON’T KNOW (88)
- REFUSED (99)

3.25 You feel safe walking in your neighborhood during the evening.

- Strongly agree (5)
- Agree (4)
- Disagree (2)
- Strongly disagree (1)
- Neither agree nor disagree (3)
- DON’T KNOW (88)
- REFUSED (99)

3.26 Your neighborhood is safe from crime.

- Strongly agree (5)
- Agree (4)
- Disagree (2)
- Strongly disagree (1)
- Neither agree nor disagree (3)
- DON’T KNOW (88)
- REFUSED (99)

3.27 Violence is a problem in your neighborhood.

- Strongly agree (5)
- Agree (4)
- Disagree (2)
- Strongly disagree (1)
- Neither agree nor disagree (3)
- DON’T KNOW (88)
- REFUSED (99)

## Neighborhood Aesthetics

3.28 There are trees along the streets in your neighborhood.

- Strongly agree (5)
- Agree (4)
- Disagree (2)
- Strongly disagree (1)
- Neither agree nor disagree (3)
- DON’T KNOW (88)
- REFUSED (99)

3.29 There are many interesting things to look at while walking in your neighborhood.

- Strongly agree (5)
- Agree (4)
- Disagree (2)
- Strongly disagree (1)
- Neither agree nor disagree (3)
- DON’T KNOW (88)
- REFUSED (99)

3.30 There are many attractive natural sights in your neighborhood, such as landscaping or views.

- Strongly agree (5)
- Agree (4)
- Disagree (2)
- Strongly disagree (1)
- Neither agree nor disagree (3)
- DON’T KNOW (88)
- REFUSED (99)

3.31 There are attractive buildings/homes in your neighborhood.

- Strongly agree (5)
- Agree (4)
- Disagree (2)
- Strongly disagree (1)
- Neither agree nor disagree (3)
- DON’T KNOW (88)
- REFUSED (99)

# DOMAIN 4: SOCIAL NETWORKS

Think of all the people you know, who know you, and who you’ve had regular contact with in the past six months. This contact could be face-to-face, by phone or mail, or on the Internet. Based on this description, how many of the following types of people do you know? Please take your time to think about this. Also, please do not count people in a particular category if you’ve already counted them in another category. For example, do not count someone as a co-worker if you’ve already counted them as a close friend. **[ENTER A NUMBER FOR EACH CATEGORY BELOW]**

4.1a Family members (immediate family, birth family, spouse, in-laws)

_____ people

- DON’T KNOW (88)
- REFUSED (99)

4.1b Close friends

_____ people

- DON’T KNOW (88)
- REFUSED (99)

4.1c Co-workers

_____ people

- DON’T KNOW (88)
- REFUSED (99)

4.1d Neighbors

_____ people

- DON’T KNOW (88)
- REFUSED (99)

4.1e People who are in your religious community or attend the same place of worship (e.g., church)

_____ people

- DON’T KNOW (88)
- REFUSED (99)

4.1f Others (e.g., people you know through recreational activities, etc.)

_____ people

- DON’T KNOW (88)
- REFUSED (99)

**[ADD THE NUMBERS ENTERED IN 4.1A – 4.1F ABOVE TO CALCULATE THE SIZE OF THE RESPONDENT’S SOCIAL NETWORK:]**

_______ SOCIAL NETWORK SIZE

Of the **[INSERT SOCIAL NETWORK SIZE]** people in your social circle:

4.2a With how many have you gone for a walk in the past week?

_____ people **[0 to SOCIAL NETWORK SIZE]**

- DON’T KNOW (88)
- REFUSED (99)

4.2b How many are likely to support you to be physically active?

_____ people **[0 to SOCIAL NETWORK SIZE]**

- DON’T KNOW (88)
- REFUSED (99)

**[FLIP TO CARD 15]**

## Social Support for Physical Activity

Please state your answers once for family and once for friends for each of the following statements. During the past three months, how often did your family or friends …

4.3 Do physical activity with you.

|  | **Never** | **Rarely** | **Sometimes** | **Often** | **Very Often** | **DON’T KNOW** | **REFUSED** |
| --- | --- | --- | --- | --- | --- | --- | --- |
| a. Family | (1) | (2) | (3) | (4) | (5) | (88) | (99) |
| b. Friends | (1) | (2) | (3) | (4) | (5) | (88) | (99) |

4.4 Offer to do physical activity with you.

|  | **Never** | **Rarely** | **Sometimes** | **Often** | **Very Often** | **DON’T KNOW** | **REFUSED** |
| --- | --- | --- | --- | --- | --- | --- | --- |
| a. Family | (1) | (2) | (3) | (4) | (5) | (88) | (99) |
| b. Friends | (1) | (2) | (3) | (4) | (5) | (88) | (99) |

4.5 Give you encouragement to do physical activity.

|  | **Never** | **Rarely** | **Sometimes** | **Often** | **Very Often** | **DON’T KNOW** | **REFUSED** |
| --- | --- | --- | --- | --- | --- | --- | --- |
| a. Family | (1) | (2) | (3) | (4) | (5) | (88) | (99) |
| b. Friends | (1) | (2) | (3) | (4) | (5) | (88) | (99) |

## Attendance of Community Courses on Physical Activity and Healthy Eating

4.6 In the past six months, have you attended any classes or participated in programs in which you engaged in physical activity, for example, a class in which you did Zumba or some other type of dance or exercise?

- YES (1)
- NO (5) 🡪 **SKIP 4.7 and GO TO 4.8**
- DON’T KNOW (88) 🡪 **SKIP 4.7 and GO TO 4.8**
- REFUSED (99) 🡪 **SKIP 4.7 and GO TO 4.8**

4.7 Please tell me which of these classes or programs you have attended in the past six months? **[ENTER UP TO THREE CLASS/PROGRAM NAME(S).]**

Class Name #1: _______________________________________

Class Name #2: _______________________________________

Class Name #3: _______________________________________

**[FLIP TO CARD 16]**

4.7a In the past six months, how frequently did you attend **[INSERT CLASS NAME #1]**?

- Once a day or more (1)
- 3-6 times a week (2)
- Once or twice a week (3)
- 2-3 times a month (4)
- Once a month (5)
- Less than once a month (6)
- DON’T KNOW (88)
- REFUSED (99)

4.7b In the past six months, how frequently did you attend **[INSERT CLASS NAME #2]**?

- Once a day or more (1)
- 3-6 times a week (2)
- Once or twice a week (3)
- 2-3 times a month (4)
- Once a month (5)
- Less than once a month (6)
- DON’T KNOW (88)
- REFUSED (99)

4.7c In the past six months, how frequently did you attend **[INSERT CLASS NAME #3]**?

- Once a day or more (1)
- 3-6 times a week (2)
- Once or twice a week (3)
- 2-3 times a month (4)
- Once a month (5)
- Less than once a month (6)
- DON’T KNOW (88)
- REFUSED (99)

4.8 In the past six months, have you attended any classes or programs that taught you about physical activity without actually engaging in physical activity during the class or program?

- YES (1) 🡪**ASK 4.9**
- NO (5) 🡪**SKIP 4.9 and GO TO 4.10**
- DON’T KNOW (88) 🡪 **SKIP 4.9 and GO TO 4.10**
- REFUSED (99) **🡪 SKIP 4.9 and GO TO 4**.**10**

4.9 Please tell me which classes or programs that taught you about physical activity you have attended in the past six months. **[ENTER UP TO THREE CLASS/PROGRAM NAME(S).]**

Class Name #1: _______________________________________

Class Name #2: _______________________________________

Class Name #3: _______________________________________

4.9a In the past six months, how frequently did you attend **[INSERT CLASS NAME #1]**?

- Once a day or more (1)
- 3-6 times a week (2)
- Once or twice a week (3)
- 2-3 times a month (4)
- Once a month (5)
- Less than once a month (6)
- DON’T KNOW (88)
- REFUSED (99)

4.9b In the past six months, how frequently did you attend **[INSERT CLASS NAME #2]**?

- Once a day or more (1)
- 3-6 times a week (2)
- Once or twice a week (3)
- 2-3 times a month (4)
- Once a month (5)
- Less than once a month (6)
- DON’T KNOW (88)
- REFUSED (99)

4.9c In the past six months, how frequently did you attend **[INSERT CLASS NAME #3]**?

- Once a day or more (1)
- 3-6 times a week (2)
- Once or twice a week (3)
- 2-3 times a month (4)
- Once a month (5)
- Less than once a month (6)
- DON’T KNOW (88)
- REFUSED (99)

4.10 Have you attended any classes or participated in programs that taught you about healthy eating in the past six months?

- YES (1) 🡪**ASK 4.11**
- NO (2) 🡪**SKIP 4.11 and GO TO DOMAIN 5**
- DON’T KNOW (88)
- REFUSED (99)

4.11 Please tell me which classes or programs on healthy eating you attended in the past six months. **[ENTER UP TO THREE CLASS/PROGRAM NAME(S).]**

Class Name #1: _______________________________________

Class Name #2: _______________________________________

Class Name #3: _______________________________________

4.11a In the past six months, how frequently did you attend **[INSERT CLASS NAME #1]**?

- Once a day or more (1)
- 3-6 times a week (2)
- Once or twice a week (3)
- 2-3 times a month (4)
- Once a month (5)
- Less than once a month (6)
- DON’T KNOW (88)
- REFUSED (99)

4.11b In the past six months, how frequently did you attend **[INSERT CLASS NAME #2]**?

- Once a day or more (1)
- 3-6 times a week (2)
- Once or twice a week (3)
- 2-3 times a month (4)
- Once a month (5)
- Less than once a month (6)
- DON’T KNOW (88)
- REFUSED (99)

4.11c In the past six months, how frequently did you attend **[INSERT CLASS NAME #3]**?

- Once a day or more (1)
- 3-6 times a week (2)
- Once or twice a week (3)
- 2-3 times a month (4)
- Once a month (5)
- Less than once a month (6)
- DON’T KNOW (88)
- REFUSED (99)

# DOMAIN 5: SOCIAL COGNITION RELATED TO PHYSICAL ACTIVITY

## Social Norms for Physical Activity

**[FLIP TO CARD 17]**

5.1 How often do you see people in your neighborhood participating in physical activity such as walking, jogging, bicycling, or playing sports?

- Very often (5)
- Often (4)
- Sometimes (3)
- Rarely (2)
- Never (1)
- DON’T KNOW (88)
- REFUSED (99)

5.2 How often do your friends participate in physical activity such as walking, jogging, bicycling, or playing sports?

- Very often (5)
- Often (4)
- Sometimes (3)
- Rarely (2)
- Never (1)
- DON’T KNOW (88)
- REFUSED (99)

5.3 How often do your family members participate in physical activity such as walking, jogging, bicycling, or playing sports?

- Very often (5)
- Often (4)
- Sometimes (3)
- Rarely (2)
- Never (1)
- DON’T KNOW (88)
- REFUSED (99)

## Goals/Intentions to Engage In Physical Activity

**[FLIP TO CARD 18]**

For the following questions, please consider physical activity as something that you do that takes at least moderate physical effort and makes you breathe a little harder than normal.

5.4 Do you intend to engage in physical activity three or more times a week for at least 10 minutes at a time during the next year?

- Definitely (5)
- Probably (4)
- Maybe (3)
- Probably not (2)
- Definitely not (1)
- DON’T KNOW (88)
- REFUSED (99)

## Barriers to Regular Physical Activity

**[FLIP TO CARD 15]**

5.5 How often are the following reasons keeping you from engaging in physical activity three or more times a week for at least 10 minutes at a time?

|  | **Never** | **Rarely** | **Sometimes** | **Often** | **Very Often** | **DON’T KNOW** | **REFUSED** |
| --- | --- | --- | --- | --- | --- | --- | --- |
| 1. Lack of interest in exercise or physical activity | (1) | (2) | (3) | (4) | (5) | (88) | (99) |
| 1. Not enough enjoyment from exercise or physical activity | (1) | (2) | (3) | (4) | (5) | (88) | (99) |
| 1. Lack of facilities in your neighborhood | (1) | (2) | (3) | (4) | (5) | (88) | (99) |
| 1. No safe places to exercise in your neighborhood | (1) | (2) | (3) | (4) | (5) | (88) | (99) |
| 1. No sidewalks in your neighborhood | (1) | (2) | (3) | (4) | (5) | (88) | (99) |
| 1. Lack of well-lit places to exercise in your neighborhood | (1) | (2) | (3) | (4) | (5) | (88) | (99) |
| 1. Too much traffic in your neighborhood | (1) | (2) | (3) | (4) | (5) | (88) | (99) |
| 1. Too dangerous to cross the street in your neighborhood | (1) | (2) | (3) | (4) | (5) | (88) | (99) |

## Self-Efficacy to Engage in Physical Activity

**[FLIP TO CARD 19]**

Please answer the following questions by rating your confidence level on a scale from 0 through 10, where 0 = I cannot do it at all, 5 = I’m moderately certain that I can do it, and 10 = I’m completely certain that I can do it.

How confident are you that you could exercise under each of the following conditions over the next six months? **[ENTER CONFIDENCE RATING FROM 0 TO 10 FOR EACH]**

5.6a. You could exercise when tired.

______ **[ENTER RATING]**

- DON’T KNOW (88)
- REFUSED (99)

5.6b. You could exercise when feeling depressed.

______ **[ENTER RATING]**

- DON’T KNOW (88)
- REFUSED (99)

5.6c. You could exercise during bad weather.

______ **[ENTER RATING]**

- DON’T KNOW (88)
- REFUSED (99)

5.6d. You could exercise when there are competing interests (like my favorite TV show).

______ **[ENTER RATING]**

- DON’T KNOW (88)
- REFUSED (99)

5.6e. You could exercise when I have a lot to do.

______ **[ENTER RATING]**

- DON’T KNOW (88)
- REFUSED (99)

5.6f. You could exercise when I don't receive support from my family and friends.

______ **[ENTER RATING]**

- DON’T KNOW (88)
- REFUSED (99)

5.6g. You could exercise when I have not exercised for a long time.

______ **[ENTER RATING]**

- DON’T KNOW (88)
- REFUSED (99)

5.6h. You could exercise when I have no one to exercise with.

______ **[ENTER RATING]**

- DON’T KNOW (88)
- REFUSED (99)

5.6i.YouI could exercise when my exercise workout is not enjoyable.

______ **[ENTER RATING]**

- DON’T KNOW (88)
- REFUSED (99)

5.6j. In general, do you believe you could engage in physical activity that makes you breathe a little harder than normal three or more times a week for at least 10 minutes at a time every week over the next six months?

______ **[ENTER RATING]**

- DON’T KNOW (88)
- REFUSED (99)

## Outcome Expectancies

**[FLIP TO CARD 20]**

This section is about physical activity, which is something you do that takes at least moderate physical effort and makes you breathe a little harder than normal. Please tell me whether you strongly disagree, disagree, agree, strongly agree, or are neutral – neither agree or disagree with the following statements.

5.7 If you participate in physical activity for three or more times a week for at least 10 minutes at a time, then …

|  | **Strongly Disagree** | **Disagree** | **Agree** | **Strongly Agree** | **Neither agree or disagree** | **DON’T KNOW** | **REFUSED** |
| --- | --- | --- | --- | --- | --- | --- | --- |
| 1. You will feel less depressed and/or bored. | (1) | (2) | (4) | (5) | (3) | (88) | (99) |
| 1. You will lose weight or get into better shape. | (1) | (2) | (4) | (5) | (3) | (88) | (99) |
| 1. You will feel less tension and stress. | (1) | (2) | (4) | (5) | (3) | (88) | (99) |
| 1. You will improve my health or reduce my risk of disease. | (1) | (2) | (4) | (5) | (3) | (88) | (99) |
| 1. You will feel more attractive. | (1) | (2) | (4) | (5) | (3) | (88) | (99) |

# DOMAIN 6: PHYSICAL HEALTH

**[FLIP TO CARD 21]**

## Physical Functioning

The following items are about activities you might do during a typical day. Please indicate how much your health now limits you in these activities.

6.1 Does your health limit you a lot, a little, or not at all when ...

|  | **Limited a lot** | **Limited a little** | **Not limited** | **DON’T KNOW** | **REFUSED** |
| --- | --- | --- | --- | --- | --- |
| 1. Doing vigorous activities, such as running, lifting heavy objects, participating in strenuous sports | (3) | (2) | (1) | (88) | (99) |
| 1. Doing moderate activities, such as moving a table, pushing a vacuum cleaner, bowling, or playing golf | (3) | (2) | (1) | (88) | (99) |
| 1. Lifting or carrying groceries | (3) | (2) | (1) | (88) | (99) |
| 1. Climbing several flights of stairs | (3) | (2) | (1) | (88) | (99) |
| 1. Climbing one flight of stairs | (3) | (2) | (1) | (88) | (99) |
| 1. Bending kneeling, or stooping | (3) | (2) | (1) | (88) | (99) |
| 1. Walking more than a mile | (3) | (2) | (1) | (88) | (99) |
| 1. Walking several blocks | (3) | (2) | (1) | (88) | (99) |
| 1. Walking one block | (3) | (2) | (1) | (88) | (99) |
| 1. Bathing or dressing yourself | (3) | (2) | (1) | (88) | (99) |

***Bodily Pain***

**[FLIP TO CARD 22]**

6.2 How much bodily pain have you had during the past four weeks?

- None (1)
- Very mild (2)
- Mild (3)
- Moderate (4)
- Severe (5)
- Very severe (6)
- DON’T KNOW (88)
- REFUSED (99)

**[FLIP TO CARD 23]**

6.3 During the past four weeks, how much did pain interfere with your normal work (including both work outside the home and housework)?

- Not at all (1)
- A little bit (2)
- Moderately (3)
- Quite a bit (4)
- Extremely (5)
- DON’T KNOW (88)
- REFUSED (99)

# DOMAIN 7: PSYCHOLOGICAL FUNCTIONING/BEHAVIORAL HEALTH

## General Psychological Distress

The next questions are about how you have been feeling during the past 30 days. For each question, indicate if you felt this way all of the time, most of the time, some of the time, a little of the time, or none of the time?

**[FLIP TO CARD 24]**

7.1a During the past 30 days, about how often did you feel nervous?

- All of the time (5)
- Most of the time (4)
- Some of the time (3)
- A little of the time (2)
- None of the time (1)
- DON’T KNOW (88)
- REFUSED (99)

7.1b During the past 30 days, about how often did you feel hopeless?

- All of the time (5)
- Most of the time (4)
- Some of the time (3)
- A little of the time (2)
- None of the time (1)
- DON’T KNOW (88)
- REFUSED (99)

7.1c During the past 30 days, about how often did you feel restless or fidgety?

- All of the time (5)
- Most of the time (4)
- Some of the time (3)
- A little of the time (2)
- None of the time (1)
- DON’T KNOW (88)
- REFUSED (99)

7.1d During the past 30 days, about how often did you feel so depressed that nothing could cheer you up?

- All of the time (5)
- Most of the time (4)
- Some of the time (3)
- A little of the time (2)
- None of the time (1)
- DON’T KNOW (88)
- REFUSED (99)

7.1e During the past 30 days, about how often did you feel that everything was an effort?

- All of the time (5)
- Most of the time (4)
- Some of the time (3)
- A little of the time (2)
- None of the time (1)
- DON’T KNOW (88)
- REFUSED (99)

7.1f During the past 30 days, about how often did you feel worthless?

- All of the time (5)
- Most of the time (4)
- Some of the time (3)
- A little of the time (2)
- None of the time (1)
- DON’T KNOW (88)
- REFUSED (99)

## Substance Use

*Binge Drinking*

7.2 Please tell me your gender. Are you:

- Male (1)
- Female (2)
- Unsure (3)
- DON’T KNOW (88)
- REFUSED (99)

**[FLIP TO CARD 25]**

7.3 On how many days in the last 30 days did you have **[IF MALE: INSERT “FIVE OR MORE”; IF FEMALE, INSERT “FOUR OR MORE”]** alcoholic drinks on the same occasion (either at the same time or within a couple of hours of each other)?

- None (0)
- 1 day (1)
- 2-3 days (2)
- About once a week (3)
- About twice a week (4)
- More than twice a week but less than most days (5)
- Most days (6)
- DON’T KNOW (88)
- REFUSED (99)

# DOMAIN 8: SOCIODEMOGRAPHIC INFORMATION

## Marital Status

**[FLIP TO CARD 26]**

8.1 What is your marital status?

**[CHOOSE ALL THAT APPLY]**

- Married (1)
- Living with a partner (2)
- Widowed (3)
- Divorced (4)
- Separated (5)
- Never married (6)
- DON’T KNOW (88)
- REFUSED (99)

## Race and Ethnicity

8.2a Are you of Hispanic, Latino or Spanish origin?

- Yes (1)
- No (5)
- DON’T KNOW/NOT SURE (88)
- REFUSED (99)

**[FLIP TO CARD 27]**

8.2b What is your race?

**[READ CHOICES IF NECESSARY. CHOOSE ALL THAT APPLY.]**

- White (1)
- Black or African American (2)
- Asian (3)
- American Indian or Alaska Native (4)
- Native Hawaiian or Other Pacific Islander (5)
- Other, specify: ______________________ (6)
- DON'T KNOW (88)
- REFUSED (99)

## Employment Status

**[FLIP TO CARD 28]**

8.3 What is your employment status?

- Employed full time (1) **→** **SKIP 8.3a and GO TO 8.4**
- Employed part time (2) **→** **SKIP 8.3a and GO TO 8.4**
- Not employed (3)
- DON’T KNOW (88) **→ SKIP 8.3a and GO TO 8.4**
- REFUSED (99) **→** **SKIP 8.3a and GO TO 8.4**

**[FLIP TO CARD 29]**

8.3a Which of the following best describes your situation?

- Looking for work (1)
- Volunteer (2)
- Student (3)
- Retired (4)
- Disabled (5)
- Other (please specify) (6) _______________________________________________________
- DON’T KNOW (88)
- REFUSED (99)

**[AFTER ASKING QUESTION 8.3a, SKIP QUESTION 8.4 AND GO TO QUESTION 8.5]**

8.4 How many hours do you work per week? _____ hours/week **[ENTER NUMBER**]

**[THIS QUESTION ASKS FOR TOTAL HOURS WORKED, AT ALL JOBS]**

- DON’T KNOW (88)
- REFUSED (99)

## Access to a Vehicle

8.5 Do you own or lease a vehicle, such as a car, van, or truck?

- Yes (1) **→ SKIP 8.6 and GO TO 8.7**
- No (5)
- DON’T KNOW (88)
- REFUSED (99)

8.6 Do you have access to a vehicle – such as a car, van, or truck - when you need one?

- Yes (1)
- No (5)
- DON’T KNOW (88)
- REFUSED (99)

## Household Income

**[BEGIN SELF-RESPONSE QUESTIONS]**

The next several questions are about your household income, food stamps, and WIC. You can answer these questions on the computer on your own, or I can ask you the questions if you prefer.

**[IF RESPONDENT SAYS THEY DO NOT WANT TO SELF-RESPOND, SAY THAT’S OK, CHECK BOX FOR QUESTIONS NOT SELF-ADMINISTERED AND PROCEED WITH ASKING THE QUESTIONS. USE RESPONSE CARDS IF NOT SELF-ADMINISTERED]**

- **Questions not self-administered**

**[IF RESPONDENT WOULD LIKE TO SELF-RESPOND:]** Before we begin this section, let me show you how to respond to these questions using the computer. **[REFER TO TRAINING PROTOCOL.]** If you have questions about what the interview question means, please ask me.

**[USE CARDS ONLY IF NOT SELF-ADMINISTERED: FLIP TO CARD 30]**

8.7 Was your total household income in 2012 $40,000 or more or less than $40,000? Household income means the combined income of everyone who lives in the house and who shares expenses and earnings.

- Less than $40,000 (1) **[DISPLAY ONLY 1-4, 88 AND 99 IN 8.8]**
- More than $40,000 (5) **[DISPLAY ONLY 5-9, 88 AND 99 IN 8.8]**
- DON’T KNOW (88)
- REFUSED (99)

**[USE CARDS ONLY IF NOT SELF-ADMINISTERED:**

**FLIP TO CARD 31 IF < $40,000 / FLIP TO CARD 32 IF > $40,000]**

8.8 What was your total household income in 2012?

- Less than $5,000 (1)
- $5,000 - $9,999 (2)
- $10,000 - $14,999 (3)
- $15,000 - $19,999 (4)
- $20,000 - $29,999 (5)
- $30,000 - $39,999 (6)
- $40,000 - $49,999 (7)
- $50,000 - $59,999 (8)
- $60,000 - $74,999 (9)
- $75,000 - $100,000 (10)
- $100,000 or more (11)
- DON’T KNOW (88)
- REFUSED (99)

8.9 Did any member of your household receive food stamps – such as SNAP, Access card, or EBT — in any of the last 12 months?

- Yes (1)
- No (5) **→ GO TO 8.10**
- DON’T KNOW (88)
- REFUSED (99)

**[EBT STANDS FOR ELECTRONIC BENEFITS TRANSFER, SNAP STANDS FOR Supplemental Nutrition Assistance Program]**

8.9a When did the household last receive food stamps?

**[RESPONDENT MAY FILL IN ANY OR ALL OF BELOW DATE FIELDS – ALL ARE NOT REQUIRED]**

____ Month

____ Year

- DON’T KNOW (88)
- REFUSED (99)

**[IF FOOD STAMPS WERE RECEIVED IN THE CURRENT OR PREVIOUS MONTH IN 8.9a, ASK 8.9a.i]**

8.9a.i What was the day your household last received a food stamp payment?

____ Day

____ Month **[confirm]**

- DON’T KNOW (88)
- REFUSED (99)

**[REFER TO CALENDARS IN RESPONSE CARD BINDER IF NEEDED]**

**[FLIP TO CARD 33]**

8.9b In **[INSERT MONTH/YEAR FROM 8.9a]**, approximately what was the total amount of food stamps received per month?

- Less than $100 (1)
- $100 - $200 (2)
- $200 - $500 (3)
- More than $500 (4)
- DON’T KNOW (88)
- REFUSED (99)

## Household Composition

8.10 How many adults other than yourself live in your household? Adults are individuals 18 years or older.

______ **[ENTER NUMBER OF ADULTS IN HOUSEHOLD OTHER THAN THE RESPONDENT.]**

- DON’T KNOW (88)
- REFUSED (99)

**[IF OTHER ADULTS RESIDE IN THE RESPONDENT’S HOUSEHOLD, ASK 8.10a. IF NO OTHER ADULTS RESIDE IN THE RESPONDENT’S HOUSEHOLD, GO TO QUESTION 8.11.]**

8.10a For each adult living in your household, please tell me their age, gender, and relationship to you.

- DON’T KNOW (88)
- REFUSED (99)

**[EXPLAIN THAT THESE ADULTS DO NOT HAVE TO BE RELATIVES – WE JUST WANT TO COUNT EVERY ADULT WHO LIVES AT THIS ADDRESS.]**

**[FILL IN FIRST ROW FOR RESPONDENT.]**

| **Age** | **Male/Female** | **Relationship** |
| --- | --- | --- |
|  |  | SELF (RESPONDENT) |
|  |  |  |
|  |  |  |
|  |  |  |
|  |  |  |
|  |  |  |
|  |  |  |
|  |  |  |

8.11 How many children live in your household? Children are individuals younger than 18 years of age.

______ **[ENTER NUMBER OF CHILDREN IN THE RESPONDENT’S HOUSEHOLD. IF NO (0) CHILDREN, SKIP 8.11a.]**

- DON’T KNOW (88)
- REFUSED (99)

**[IF CHILDREN ARE LIVING IN THE RESPONDENT’S HOUSEHOLD, ASK 8.11A. IF NO CHILDREN ARE LIVING IN THE RESPONDENT’S HOUSEHOLD, BEGIN DOMAIN 9.]**

8.11a For every child living in your household, please tell me their age, gender and relationship to you.

- DON’T KNOW (88)
- REFUSED (99)

**[EXPLAIN THAT THESE CHILDREN DO NOT HAVE TO BE RELATIVES – WE JUST WANT TO COUNT EVERY CHILD WHO LIVES AT THIS ADDRESS.]**

| **Age** | **Male/Female** | **Relationship** |
| --- | --- | --- |
|  |  |  |
|  |  |  |
|  |  |  |
|  |  |  |
|  |  |  |
|  |  |  |
|  |  |  |
|  |  |  |

# DOMAIN 9: PHRESH QUESTIONS

## Current Major Food Shopping Locations

9.1 What is the name and address of the main store where you most often do your major food shopping?

**[IF THE RESPONDENT HAS TROUBLE IDENTIFYING THE STORE, REFER THEM TO THE LIST AT THE BACK OF THE RESPONSE BINDER. IF THE STORE IS NOT ON THE LIST OF STORES PROVIDED, GET WHATEVER IDENTIFYING INFORMATION THE RESPONDENT CAN PROVIDE.]**

**[IF THERE ARE TWO STORES THE RESPONDENT USES EQUALLY, ASK THE RESPONDENT TO CHOOSE ONE STORE NOW. RESPONDENTS WILL BE ABLE TO ADD THE SECOND STORE IN THE NEXT SET OF QUESTIONS.]**

Name and location of store:

_______________________________________________________

- Other, specify name of store: ____________________________
- DON’T KNOW (88)
- REFUSED (99)

**[IF ANSWER TO 9.1 IS OTHER, ASK 9.1a.]**

9.1a Where is the **[INSERT NAME OF STORE IN 9.1]**? Do you know its address or cross streets?

**[TRY TO GET AS MUCH IDENTIFYING INFORMATION AS POSSIBLE.]**

Address or cross streets of store: ________________________________________________________

- DON’T KNOW (88)
- REFUSED (99)

9.2 How many times did you visit [FILL IN NAME OF STORE FROM 9.1] for major food shopping in the past month? **[DO NOT READ THE RESPONSE CHOICES. ALLOW THE RESPONDENT TO ANSWER AND RECORD THEIR RESPONSE.]**

- Never (0)
- Once (1)
- Twice (2)
- Three times (3)
- Four times (4)
- Five times (5)
- Six times (6)
- Seven times (7)
- Eight times (8)
- More than eight times (9)
- DON’T KNOW (88)
- REFUSED (99)

**[FLIP TO CARD 34]**

9.3 During the past month, how many other stores have you visited for major food shopping?

- None (1) **→ GO TO 9.4**
- One (2)
- Two (3)
- Three (4)
- Four (5)
- Five (6)
- More than 5 (7)
- DON’T KNOW (88)
- REFUSED (99)

9.3a What is the name and location of the other store you have visited most often for major food shopping during the past month?

**[FLIP TO LIST OF STORES IF RESPONDENT HAS TROUBLE WITH THE NAME OR LOCATION OF THE STORE]**

Name and location of store:

_______________________________________________________

- Other, specify name of store: ____________________________
- DON’T KNOW (88)
- REFUSED (99)

**[IF ANSWER TO 9.3a IS OTHER, ASK 9.3b.]**

9.3b Where is the **[INSERT NAME OF STORE IN 9.3a]**? Do you know the address or cross streets?

**[TRY TO GET AS MUCH IDENTIFYING INFORMATION AS POSSIBLE.]**

Address or cross streets of store: ________________________________________________________

- DON’T KNOW (88)
- REFUSED (99)

**[FLIP TO CARD 35]**

## Reasons for Choice of Major Food Shopping Location

9.4 If you were to choose just one main reason that you chose to do your major food shopping at **[INSERT NAME OF STORE CITED IN 9.1]**, which of the following would you choose?

Name of store:

_______________________________________________________

- DON’T KNOW (88)
- REFUSED (99)

**[READ LIST OF OPTIONS AND RECORD ONLY ONE OPTION. IF RESPONDENT ENDORSES MORE THAN ONE, ASK THEM TO PICK THE ONE THAT IS MOST IMPORTANT IN CAUSING THEN TO SHOP AT THE STORE WHERE THEY DO THEIR MAJOR FOOD SHOPPING.]**

- Quality of food (1)
- Price (2)
- Choice of items (3)
- Convenience of store location (4)
- Customer service (5)
- Cleanliness of the store (6)
- DON’T KNOW (88)
- REFUSED (99)

9.5a What is the name and address of the grocery store that is closest to your home?

Name and location of store:

_______________________________________________________

- Other, specify name of store: ____________________________
- DON’T KNOW (88)
- REFUSED (99)

**[IF THE RESPONDENT HAS TROUBLE IDENTIFYING THE STORE, REFER THEM TO THE LIST AT THE BACK OF THE RESPONSE BINDER. IF THE STORE IS NOT ON THE LIST OF STORES PROVIDED, GET WHATEVER IDENTIFYING INFORMATION THE RESPONDENT CAN PROVIDE. IF THE RESPONDENT IS NOT CERTAIN WHICH STORE IS CLOSEST, ASK THEM TO SAY WHICH STORE THEY THINK IS THE CLOSEST, REGARDLESS OF WHETHER OR NOT IT ACTUALLY IS THE CLOSEST.]**

**[IF THE STORE NAMED IN 9.5a IS DIFFERENT FROM THE STORE NAMED IN 9.1, ASK 9.5b; IF THE STORE NAMED IN 9.5a IS THE SAME STORE NAMED IN 9.1, SKIP TO 9.6.]**

9.5b You told us that the grocery store closest to your home is **[INSERT NAME OF STORE FROM 9.5a]**, but that you do most of your food shopping at **[INSERT NAME OF STORE FROM 9.1]**. What is the main reason that you shop at a store that is farther away from your home?

- Quality of food (1)
- Price (2)
- Choice of items (3)
- Convenience of store location (4)
- Customer service (5)
- Cleanliness of the store (6)
- DON’T KNOW (88)
- REFUSED (99)

**[READ LIST OF OPTIONS AND RECORD ONLY ONE OPTION. IF RESPONDENT ENDORSES MORE THAN ONE, ASK THEM TO PICK THE ONE THAT IS MOST IMPORTANT IN CAUSING THEN TO SHOP AT A STORE THAT IS NOT THE ONE CLOSEST TO THEIR HOME.]**

## Perceived Influence of Advertisement of Price on Choice of Food Shopping Location

**[FLIP TO CARD 36]**

9.6 In the past month, how often did you decide to do your major food shopping at a store because you had seen advertisements or flyers showing that this store had the lowest food prices?

- Never (1)
- Occasionally (2)
- Sometimes (3)
- Often (4)
- DON’T KNOW (88)
- REFUSED (99)

## Timing of Food Shopping in Relation to Receipt of Food Stamps

**[ASK 9.7 ONLY OF RESPONDENTS WHO REPORTED THAT THEY HAVE RECEIVED SNAP BENEFITS (I.E., PUBLIC ASSISTANCE) IN THE PAST MONTH IN RESPONSE TO QUESTION 8.9 ABOVE.]**

9.7 Do you tend to do most of your major food shopping soon after you get SNAP benefits (food stamps)?

- YES (1)
- NO (5)
- DON’T KNOW (88)
- REFUSED (99)

## Modes of Transportation Used to Travel to and from Major Food Shopping Location

9.8 When you do your major food shopping, how often do you take each of the following modes of transportation to get from your home to the store where you do your major food shopping?

| **Mode of Transportation** | **Never** | **Occasionally** | **Sometimes** | **Often** | **DON’T KNOW** | **REFUSED** |
| --- | --- | --- | --- | --- | --- | --- |
| a. Drive your vehicle | (1) | (2) | (3) | (4) | (88) | (99) |
| b. Borrow a friend or family member’s vehicle | (1) | (2) | (3) | (4) | (88) | (99) |
| c. Jitney | (1) | (2) | (3) | (4) | (88) | (99) |
| d. Walk | (1) | (2) | (3) | (4) | (88) | (99) |
| e. Public transportation | (1) | (2) | (3) | (4) | (88) | (99) |
| f. Bicycle | (1) | (2) | (3) | (4) | (88) | (99) |
| g. Get a ride | (1) | (2) | (3) | (4) | (88) | (99) |
| h. Carpool | (1) | (2) | (3) | (4) | (88) | (99) |
| i. Taxi | (1) | (2) | (3) | (4) | (88) | (99) |
| j. ACCESS van | (1) | (2) | (3) | (4) | (88) | (99) |
| k. Social service vehicle | (1) | (2) | (3) | (4) | (88) | (99) |
| l. Other, please specify: | (1) | (2) | (3) | (4) | (88) | (99) |

9.9 When you do your major food shopping, how often do you take each of the following modes of transportation for the return trip from the store to your home?

| **Mode of Transportation** | **Never** | **Occasionally** | **Sometimes** | **Often** | **DON’T KNOW** | **REFUSED** |
| --- | --- | --- | --- | --- | --- | --- |
| a. I drive my vehicle | (1) | (2) | (3) | (4) | (88) | (99) |
| b. I borrow a friend or family member’s vehicle | (1) | (2) | (3) | (4) | (88) | (99) |
| c. Jitney | (1) | (2) | (3) | (4) | (88) | (99) |
| d. Walk | (1) | (2) | (3) | (4) | (88) | (99) |
| e. Public transportation | (1) | (2) | (3) | (4) | (88) | (99) |
| f. Bicycle | (1) | (2) | (3) | (4) | (88) | (99) |
| g. Get a ride | (1) | (2) | (3) | (4) | (88) | (99) |
| h. Carpool | (1) | (2) | (3) | (4) | (88) | (99) |
| i. Taxi | (1) | (2) | (3) | (4) | (88) | (99) |
| j. ACCESS van | (1) | (2) | (3) | (4) | (88) | (99) |
| k. Social service vehicle | (1) | (2) | (3) | (4) | (88) | (99) |
| l. Other, please specify: | (1) | (2) | (3) | (4) | (88) | (99) |

## Transit Time to and from Major Food Shopping Location

When you think about the total time it takes to shop, can you break it into time for getting there and back and time for shopping?

9.10a How long does it take just for travel to the store and back?

______ hours _____ minutes

- DON’T KNOW (88)
- REFUSED (99)

9.10b How long does it take to complete your shopping once you are there?

______ hours _____ minutes

- DON’T KNOW (88)
- REFUSED (99)

## Usage of Food Banks

**[FLIP TO CARD 37]**

9.11 How many times during the past year did you get food or other household goods from a food pantry or food bank? **[DO NOT GIVE EXAMPLES FOR RESIDENTS OF OTHER NEIGHBORHOODS. IF RESPONDENT ASKS YOU TO DEFINE FOOD PANTRY OR BANK, GIVE A FEW EXAMPLES: IN HILL DISTRICT: BETHEL AME MISSIONARY SOCIETY FOOD PANTRY AND JUBLILEE KITCHEN; IN HOMEWOOD OR LARIMER: BETHANY BAPTIST CHURCH FOOD PANTRY AND PRODUCE TO PEOPLE.]**

- Never (0) **→ GO TO 9.14**
- Less than once a month (1)
- Once a month (2)
- Twice a month (3)
- Three times a month (4)
- Once a week (5)
- Twice a week (6)
- Three times a week (7)
- More than three times a week (8)
- DON’T KNOW (88)
- REFUSED (99)

9.11a When was the last time you got food from a food pantry or food bank?
**[REFER TO THE CALENDAR IN YOUR RESPONSE CARD BINDER IF NEEDED.]**

______ / ______ / ______ **[RECORD DATE IN MM/DD/YY FORMAT]**

- DON’T KNOW (88)
- REFUSED (99)

**[SKIP 9.12 FOR RESIDENTS OF OTHER NEIGHBORHOODS.]**

9.12 I will read you a list of local food banks. For each food bank, please tell me whether or not you got food or other household goods from there in the past year.

| **[HILL DISTRICT: FLIP TO CARD 38]** | **Yes** | **No** | **DON’T KNOW** | **REFUSED** |
| --- | --- | --- | --- | --- |
| 1. Bedford Dwellings Food Bank | (1) | (5) | (88) | (99) |
| 1. Bethel AME Missionary Society Food Pantry | (1) | (5) | (88) | (99) |
| 1. Calvary Baptist Church Food Pantry & Soup Kitchen | (1) | (5) | (88) | (99) |
| 1. Central Baptist Church Soup Kitchen | (1) | (5) | (88) | (99) |
| 1. Jubilee Kitchen Food Pantry (Polish Hill and/or Lower Hill District) | (1) | (5) | (88) | (99) |
| 1. Produce to the People | (1) | (5) | (88) | (99) |
| 1. K. Leroy Irvis Tower Food Bank | (1) | (5) | (88) | (99) |
| 1. The Legacy Food Bank | (1) | (5) | (88) | (99) |
| 1. Monumental Mission Ministries Food Pantry | (1) | (5) | (88) | (99) |
| 1. New Light Baptist Temple Church | (1) | (5) | (88) | (99) |
| 1. Oak Hill Food Bank | (1) | (5) | (88) | (99) |
| 1. St. Benedict the Moor Food Pantry | (1) | (5) | (88) | (99) |
| 1. Other, specify: __________________________ | (1) | (5) | (88) | (99) |

| **[HOMEWOOD OR LARIMER: FLIP TO CARD 39]** | **Yes** | **No** | **DON’T KNOW** | **REFUSED** |
| --- | --- | --- | --- | --- |
| aa. Bethany Baptist Church Food Pantry | (1) | (5) | (88) | (99) |
| bb. BTC Center, Inc. Food Pantry | (1) | (5) | (88) | (99) |
| cc. Good Samaritan Church of God in Christ Food Pantry | (1) | (5) | (88) | (99) |
| dd. Mt. Ararat Baptist Church | (1) | (5) | (88) | (99) |
| ee. Nazarene Baptist Church Food Pantry | (1) | (5) | (88) | (99) |
| ff. Produce To People | (1) | (5) | (88) | (99) |
| gg. Salvation Army of Homewood Crisis Pantry | (1) | (5) | (88) | (99) |
| hh. Shiloh Community Missionary Baptist Church Soup Kitchen | (1) | (5) | (88) | (99) |
| ii. St. Charles Lwanga Food Pantry | (1) | (5) | (88) | (99) |
| jj. YMCA Homewood Food Pantry | (1) | (5) | (88) | (99) |
| kk. Other, specify: ___________________________ | (1) | (5) | (88) | (99) |

**[FLIP TO CARD 40]**

**[IF RESPONDENT NAMED MORE THAN ONE FOOD BANK, CHOOSE ALL ITEMS RECEIVED AT ANY OF THE FOOD BANKS.]**

9.13 Which of the following items did you get from a food bank in the past year?

- Fresh fruits (1)
- Fresh vegetables (2)
- Low-fat foods (3)
- Whole grain foods (4)
- Canned goods (5)
- Meat (6)
- Snack foods (sweet or salty) (7)
- Soda (8)
- Household goods (9)
- Meals (10)
- Other, specify: ___________________(11)
- DON’T KNOW (88)
- REFUSED (99)

**[FLIP TO CARD 37]**

## Consumption of Meals in Restaurants in Which Access to Food Is Unlimited

9.14 In the past year, how often have you eaten a meal at a restaurant that offers access to unlimited food during your visit for one price, such as an “all-you-can-eat” buffet?

- Never (0)
- Less than once a month (1)
- Once a month (2)
- Twice a month (3)
- Three times a month (4)
- Once a week (5)
- Twice a week (6)
- Three times a week (7)
- More than three times a week (8)
- DON’T KNOW (88)
- REFUSED (99)

# DOMAIN 10: HEIGHT AND WEIGHT MEASUREMENT (BODY MASS INDEX)

**[MEASURE HEIGHT AND WEIGHT ONLY IF RESPONDENT HAS PREVISOUSLY CONSENTED TO BE MEASURED. IF RESPONDENT DECLINED TO BE MEASURED, ASK RESPONDENT TO SELF-REPORT HEIGHT AND WEIGHT AND RECORD. REFER TO TRAINING MATERIALS FOR PROTOCOL TO MEASURE RESPONDENT HEIGHT AND WEIGHT.]**

Next I would like to measure your weight and height. First, can you estimate your weight?

**[IF RESPONDENT’S EXTIMATED WEIGHT EXCEEDS THE CAPACITY OF THE SCALE (400 LBS), EXPLAIN THAT WE ARE NOT ABLE TO GET AN ACCURATE MEASUREMENT ON OUR SCALE SO WE WILL USE THEIR SELF-REPORT. CHECK THE BOX FOR “SCALE INSUFFICIENT FOR RESPONDENT WEIGHT” AND FILL IN SELF-REPORTED WEIGHT. CONTINUE TO MEASURE HEIGHT UNLESS RESPONDENT HAS REFUSED.]**

10.1a Measured Weight **__________** (lbs) **[RECORD WEIGHT TO 0.01 POUND]**

- CHECK THIS BOX IF SCALE INSUFFICIENT FOR RESPONDENT WEIGHT
  **[USE SELF-REPORT]**
- CHECK THIS BOX IF WEIGHT IS SELF-REPORTED

10.1b Height **_________** (inches) **[RECORD HEIGHT TO 1/8 OF AN INCH]**

- CHECK THIS BOX IF MEASURED HEIGHT IS ADJUSTED
- CHECK THIS BOX IF HEIGHT IS SELF-REPORTED

COMMENTS:

## Activity Meter Instructions

Before I explain more about the activity meter and activity diary, I want to ask if you have any plans to go out of town or engage in any kind of irregular activity over the next 7 days? I am asking because we would like for you to wear this activity meter and fill out the diary for the next 7 days in a row while going about your daily routine as usual. If you are out of town or plan to do something you do not normally do, then this will interfere with what we are trying to look at. An example of an irregular activity is undergoing a medical procedure. Do you have travel or irregular activities planned over the next 7 days? **[IF PARTICIPANT DOES HAVE TRAVEL OR IRREGULAR ACTIVITIES PLANNED THAT INTERFERE WITH THIS TASK, THEN SAY:]** Thank you for letting me know. I will come to your home when you are back to your regular schedule **[SET UP A TIME; IDEALLY THE DAY AFTER THEY ARE BACK TO THEIR REGULAR SCHEDULE]** to show you how to wear the activity meter and fill out the activity diary and will explain them and the receipt collection task then. I will call you that day as a reminder. **[BE SURE TO RECORD PARTICIPANT’S PHONE NUMBER. MOVE ON TO BMI FEEDBACK SECTION.]

[IF PARTICIPANT DOES NOT HAVE ANY TRAVEL OR IRREGULAR ACTIVITIES PLANNED, THEN GO OVER KEY POINTS:]** Okay, great. Now I’m going to tell you more about the activity meter, activity diary, and receipt collection tasks. First, I’ll explain the activity meter and show you how to wear it. Then I’ll explain how to fill out the activity diary and last I’ll explain how to collect receipts.

**[IF NEEDED, REFER TO THE DOCUMENT “CHECKLIST FOR ACTIVITY METER DELIVERY*”* AS A GUIDE WHEN EXPLAINING THE METER TO THE PARTICIPANT.]**

Because this study is interested in physical activity, we are asking you to wear this device that measures your movement. It is called an activity meter. This meter is not a tracking device – we can’t tell what you are doing or where you are. It does not measure heart rate. It is safe and should not cause discomfort when worn. You wear it on a band around your wrist. We’d prefer you wear it on the wrist of your non-dominant arm, which is the arm you don’t write with. The meter should not be covered by your clothing. Before I leave, we can put the meter on together and then you can just leave it on. You don’t have to turn the meter on or off – it will run automatically. It’s important that you wear the meter for the next 7 days in a row. Just go about your regular activities while wearing the meter – you don’t have to do anything special while you are wearing it. The meter is just for you – don’t let anyone else wear it or handle it. Please don’t unscrew, take apart, or attempt to fix the meter yourself. The meter is only for research; it has no monetary value. While you are wearing the activity meter, we will ask you to complete a daily activity diary in order for us to better understand how you travel from place to place. We will also ask you to save all of your food and beverage receipts for the same 7 days to learn more about where you shop and the foods you buy. I will provide you with more instructions on these last two tasks separately, after we go over the activity meter.

You will wear the activity meter, and fill out the activity diary, which I’ll explain in a minute, for 7 consecutive days, day and night, starting today **[REFER TO CALENDAR FOR TODAY’S DATE]**. Please wear the meter for 7 full days – do NOT take it off while you are sleeping. You can also wear the meter while you shower. You can return your meter on **[INSERT DAY AND DATE.]**

**[WRITE DAY AND DATE PARTICIPANT CAN RETURN ON THE PARTICIPANT’S INFORMATION SHEET AND** **HAND IT TO THE PARTICIPANT.]**

Here is an information sheet and some frequently asked questions. I’ll let you read this over while I activate the meter. Then we can discuss any questions you have and I’ll show you how to wear the meter.

**[INITIALIZE THE ACCELEROMETER WHILE THE PARTICIPANT REVIEWS THE INFORMATION SHEET:**

- **ENTER PHRESH PLUS ID IN THE FIELD FOR “SUBJECT NAME”. DO NOT ENTER THE PARTICIPANT’S NAME.**
- **ENTER PARTICIPANTS GENDER, RACE, HEIGHT AND WEIGHT. ASK THEIR RACE AGAIN IF NEEDED**
- **ENTER “WRIST” IN THE FIELD FOR LIMB**
- **ASK PARTICIPANT WHICH IS THEIR NON-DOMINANT ARM AND ENTER ACCORDINGLY.**

**FILL OUT THE FIELD REPORT:**

- **ACTIVITY METER SERIAL NUMBER**
- **CHECK THE BOX FOR LEFT OR RIGHT WRIST**
- **CHECK THE BOX FOR NON-DOMINANT OR DOMINANT ARM**
- **DATE DELIVERED**
- **FIRST FULL DAY OF WEAR.]**

Okay, the meter is now turned on. Wearing the meter may take a day or two to get used to. You need to wear the meter around your wrist using this band, with the meter facing upward, just like wearing a watch**. [SHOW PARTICIPANT HOW TO PUT THE METER ON AND TAKE IT OFF, AND HELP THEM ADJUST THE BAND.**

Great, you can just leave it on until **[INSERT DATE OF PICKUP].** Do not remove it at any time. The meter is waterproof, so you can wear it when you shower or bathe; however, if you wish to keep the band from getting wet, you can remove the meter to shower then put it right back on again when done. The meter should not be covered by your clothing when you are wearing it. It is very important that you wear the meter around your wrist. Please do not wear it around your upper arm or leg. Please do not put it in your pocket or in your bag or purse.

Do you have any questions about wearing the activity meter?

**[IF PARTICIPANT HAS QUESTIONS, ANSWER THEM. THEN EXPLAIN THE ACTIVITY DIARY.]**

**[ACTIVITY DIARY:]** Now let’s take a look at the activity diary. We are asking that you fill out this diary during the 7 days that you wear the meter. There are two double-sided sheets for each day to make sure you have enough space. The first page has instructions for how to fill it out; let’s go over the instructions together.

**[WRITE THE PHRESH PLUS ID ON EACH PAGE OF THE ACTIVITY DIARY. ALSO WRITE THE DATE FOR EACH DAY THE PARTICIPANT WILL FILL OUT THE DIARY.]**

**[HAND PARTICIPANT THE ACTIVITY DIARY AND ASK THEM TO LOOK AT THE INSTRUCTIONS.]** Please fill out one row for every place you go on that day, including where you were at the beginning of your day. For each day, use one row for each place you go to; check the most appropriate box, or write in the best answer, and remember to complete the Sleep Notes section at the top of the grid.

**[FLIP PAGE TO LOOK AT THE EXAMPLE:]** First, tell us where you started your day – at home or somewhere else – and write the time you left this place. Circle AM or PM. You do not need to tell us the name of the place or the address. Then, fill out the first two sleep questions: tell us what time you woke up that morning, and rate the quality of your sleep for the previous night. One (1) means your sleep was very bad and you weren’t well rested and five (5) means your sleep was very good and you slept soundly and feel well rested. You’ll fill out the rest of the sleep questions at the end of the day.

Next, tell us where you went next – you’ll do the same thing for every place you go. Check the most appropriate box under Type of Place or write in the better answer under “Other.” You do not need to tell us the address of anyplace you go or anyone’s name if you go to someone else’s house. If you went to a park, store, or exercise facility, such as a gym or aerobics class, tell us the name of that place. For example, a name of an exercise facility might be the Hill District YMCA. Again, you do not need to tell us the address. Also check the box for whether this place was inside the Hill/Homewood or Larimer or outside the Hill/Homewood or Larimer. Next, write the time you arrived and check the box for how you got there.

Next, check the box that corresponds with your activity level most of the time you were at that place. There are three activity levels to choose from: Sedentary means that you weren’t moving very much; you spent most of the time standing, sitting, or lying down. Moderate means that you were moving around some, such as stretching or walking. Vigorous means that you were moving around a lot — doing more than walking, such as running, dancing, taking an aerobics class, doing heavy housework, or playing sports. We know it’s possible to do several levels of activity at one place — for example, if you are at home on the weekend, you might be sedentary when you are watching TV but moderate if you are doing light housework – check the box that indicates how active you were **most of the time** you were in that place. Finally, write down when you left. Circle AM or PM.

At the end of the day, go back to the top of the page to answer the rest of the sleep questions. Check the box to indicate if you took a nap during the day. If you did nap, write down the time. Circle AM or PM. Next, check the box to indicate if you took any medication to help you fall asleep that night. Please do not tell us what medication it was. Last, write down the time you went to bed. Circle AM or PM.

Do you have any questions about the activity diary? **[IF SO, ANSWER THEM BEFORE MOVING ON.]**

**[RECEIPTS:]** Now I’m going to tell you more about the food and beverage receipt collection task and give you some materials. Because we are interested in diet, health, and the neighborhood environment, as well as physical activity habits, we are asking you to save all receipts of any food purchases. This includes snacks, meals, drinks, and grocery shopping. We’re asking you to do this for one full week; that is, for the next 7 days in a row, starting tomorrow. You will return the receipts on the same day you return the activity meter and activity diary – **[INSERT DATE OF PICKUP]**.

**[WRITE DATE OF PICKUP ON THE “FOOD RECEIPT COLLECTION INFORMATION SHEET” AND HAND IT TO THE PARTICIPANT.]**

Here is an information sheet with more details about collecting food and beverage receipts and some frequently asked questions. **[REFER TO SHEET:]** On this sheet is a list of some places to save receipts from, like grocery stores, supermarkets, food stands, convenience and neighborhood stores, gas stations, restaurants, and bars. Please save any receipt from a food or beverage purchase, no matter where or what time of day that purchase was made. **[POINT OUT WHERE THIS LIST IS ON THE SHEET].** If you wish, you can black out any non-food or drink items on the receipt. If the place where you obtained the receipt does not include the date or name of the store, please write those two things on the receipt itself. As you collect food and beverage receipts throughout the week, place them in this envelope. Do you have any questions?

**[WRITE PHRESH PLUS ID AND DATES OF COLLECTION ON ENVELOPE AND GIVE IT TO THE PARTICIPANT].**

I will call you tomorrow to answer any questions you may have. Can you please give me your phone number and a couple of good times to call?

**[WRITE RESPONDENT’S PHONE NUMBER ON FIELD REPORT; WRITE GOOD TIMES TO CALL IN THE NOTES SECTION OF THE FIELD REPORT.]**

If you have any questions or problems with your meter in the next 7 days, call the Field Office at 412.281.4600. The Field Office number is at the bottom of your information sheet **[POINT OUT WHERE THE NUMBER IS].**

**[NOW GO OVER RETURNING THE ACTIVITY METER, DIARY AND RECEIPTS:]** When you return the meter, activity diary, and food receipts, you will receive a $35 debit gift card to thank you for participating. I will call you when you are finished wearing the meter and we can arrange a time when I can pick it up. Or, you can return the meter, activity diary, and food receipts to the Field Office, which is located in the Hill District.

# CONCLUSION

## Gift Card Preference

Thank you very much for your time and for participating in this interview. As a reminder, all the information you provided will be kept confidential. You will receive $25 for your participation today, and $35 for wearing the activity meter. The payments are in the form of a debit gift card – the kind that can be used anywhere. Is it okay if we mail these to you?

**[IF YES, CONFIRM NAME AND ADDRESS.]**

Name _____________________________________________

Address ___________________________________________

**[IF NO, READ FOLLOWING:]** You can pick up the gift card from our Field Office, which is located in The Blakey Building, 1908 Wylie Avenue in the Hill District. Please remember to bring picture ID when you come to pick up your gift card. **[IF RESPONDENT DOES NOT HAVE A PICTURE ID, ASK THEM TO BRING AN ID WITH THEIR NAME AND ADDRESS.]** Please call the PHRESH Field Office at 412.281.4600 to arrange a time to pick up your gift card during normal business hours and ask to speak with La’Vette Wagner. .

## Contact Information of Two Friends or Relatives

As I mentioned before, we are going to contact you and ask you to complete this interview again in two years - you’d get another $60 - and we’d like to make sure we can find you. So, I’d also like to ask you for the name and contact information – phone number or email – of two friends or relatives who could help us find you again in two years, if we need to.

**[IF RESPONDENT REFUSES, ENTER NONE FOR NAME AND EMAIL.]**

|  | **1st Contact** |  | **2nd Contact** |
| --- | --- | --- | --- |
| Name: |  |  |  |
| Email: |  |  |  |
| Phone: |  |  |  |

## Height, Weight, and BMI Feedback

**[CONSULT THE BMI CALCULATION CHART AND WRITE DOWN HEIGHT, WEIGHT AND BMI ON THE APPROPRIATE FEEDBACK FORM. WHEN CONSULTING THE BMI TABLE, ROUND HEIGHT TO THE NEAREST INCH. THERE IS ONE FORM FOR RESPONDENTS WHO TOLD YOU THEIR HEIGHT AND WEIGHT (SELF-REPORT) AND ANOTHER FORM FOR THOSE WHO HAD THEIR HEIGHT AND WEIGHT MEASURED. FOLD THE BMI FEEDBACK FORM AND PLACE IT IN A WHITE BUSINESS ENVELOPE.]**

Now I would like to give you the results of your height and weight and BMI**. [HAND RESPONDENT THE ENVELOPE WITH THEIR COMPLETED FORM. IF RESPONDENT HAS QUESTIONS ABOUT WHAT IS ON THE FORM, REPLY:]** I don’t know anything more than what is on the form. If you have questions, I suggest you ask your health care provider. **[IF RESPONDENT PERSISTS, SAY:]** I’m sorry but I’m not qualified to provide any other information. If you have questions, I suggest you ask your health care provider.

Thank you very much for participating in PHRESH *Plus*!
